# Preclinical Evaluation of a PSMA-Targeting Homodimer with an Optimized Linker for Imaging of Prostate Cancer

**DOI:** 10.3390/molecules28104022

**Published:** 2023-05-11

**Authors:** Erika Murce, Savanne Beekman, Evelien Spaan, Maryana Handula, Debra Stuurman, Corrina de Ridder, Yann Seimbille

**Affiliations:** 1 Department of Radiology and Nuclear Medicine, University Medical Center Rotterdam, Erasmus MC, 3015 GD Rotterdam, The Netherlands; e.murcesilva@erasmusmc.nl (E.M.); s.beekman@erasmusmc.nl (S.B.); e.spaan@erasmusmc.nl (E.S.); m.handula@erasmusmc.nl (M.H.); d.stuurman@erasmusmc.nl (D.S.); c.deridder@erasmusmc.nl (C.d.R.); 2 Erasmus MC Cancer Institute, 3015 GD Rotterdam, The Netherlands; 3 Life Sciences Division, TRIUMF, Vancouver, BC V6T 2A3, Canada

**Keywords:** bivalent agent, ligand optimization, preclinical evaluation, prostate cancer, prostate-specific membrane antigen, radiopharmaceutical design, SPECT/CT

## Abstract

Prostate-specific membrane antigen (PSMA) targeting radiopharmaceuticals have been successfully used for diagnosis and therapy of prostate cancer. Optimization of the available agents is desirable to improve tumor uptake and reduce side effects to non-target organs. This can be achieved, for instance, via linker modifications or multimerization approaches. In this study, we evaluated a small library of PSMA-targeting derivatives with modified linker residues, and selected the best candidate based on its binding affinity to PSMA. The lead compound was coupled to a chelator for radiolabeling, and subject to dimerization. The resulting molecules, **22** and **30**, were highly PSMA specific (IC_50_ = 1.0–1.6 nM) and stable when radiolabeled with indium-111 (>90% stable in PBS and mouse serum up to 24 h). Moreover, [^111^In]In-**30** presented a high uptake in PSMA expressing LS174T cells, with 92.6% internalization compared to 34.1% for PSMA-617. Biodistribution studies in LS174T mice xenograft models showed that [^111^In]In-**30** had a higher tumor and kidney uptake compared to [^111^In]In-PSMA-617, but increasing T/K and T/M ratios at 24 h p.i. Tumors could be clearly visualized at 1 h p.i. by SPECT/CT after administration of [^111^In]In-**22** and [^111^In]In-PSMA-617, while [^111^In]In-**30** showed a clear signal at later time-points (e.g., 24 h p.i.).

## 1. Introduction

Prostate carcinoma is the second most common cancer in men, with 1,400,000 new cases and 375,000 deaths worldwide in 2020 [1]. Despite having a good prognosis at early stages, patients with advanced prostate cancer (stage IV) often develop castration resistance and metastases. Novel emerging therapeutic approaches, such as boron neutron capture therapy [2,3], photodynamic therapy [4], immunotherapy [5], and radioligand therapy, are showing promising results and gaining increasing interest in the field. Radioligand therapy (RLT) targeting the prostate-specific membrane antigen (PSMA) has recently emerged as a promising treatment modality. Thus, Pluvicto^TM^ (formerly [^177^Lu]Lu-PSMA-617) and its diagnostic counterpart Locametz^TM^ (formely [^68^Ga]Ga-PSMA-11) received FDA approval following their clinical validation [6,7]. Furthermore, several clinical studies showed promising results on combination treatment of PSMA-mediated RLT with other therapeutic modalities [8,9,10] and when RLT is integrated at an earlier-stage of the disease [11,12,13]. 

However, a significant subset of patients still progress after therapy [14,15,16], while patients presenting low intensity PSMA uptake show worse prognosis [17]. Moreover, the choice of diagnostic companion may influence the eligibility for Lu-PSMA treatment. There is also no consensus on the choice of the agent, particularly regarding the choice or combination between FDG-PET and PSMA-PET [18]. Recommendations for improvement of therapy [19] include, amongst others, pre-therapy personalized screening of patients [20,21,22], personalized dosimetry plans [23], combination regimes including both [^177^Lu]Lu-PSMA and [^225^Ac]Ac-PSMA [24,25], and ligand optimization [26].

Structural optimization aims to improve the therapeutic efficacy of PSMA-targeting agents by reducing the radiation dose to non-targeted organs, while retaining or increasing the dose delivered to the tumor. The kidneys and the salivary glands are the dose-limiting organs in the treatment with Lu-PSMA. The most common strategy is the modification of the linker region, which can significantly affect the pharmacokinetics, binding, and biodistribution of the radiotracer [27]. Considerable advances on rational ligand design have taken place over the last decades, which was possible due to the structural characterization of PSMA [28,29,30]. Glutamate–urea–lysine-targeting vectors were first introduced as peptidomimetics based on the structure of NAAG (N-acetyl-L-aspartyl-L-glutamate), a neurotransmitter which is cleaved by the glutamate carboxypeptidade II (also known as PSMA) into *N*-acetylaspartate and glutamate [31,32,33,34]. The catalytic site of PSMA can be divided in three major parts, namely, the catalytic site containing a S1’ glutamate recognition pocket, a Zn^2+^ active site, and a deep funnel of 20 Å extending from the zinc(II) atom to the external surface of the protein [28]. The urea moiety interacts with the zinc, while the carboxylate groups from the glutamate and lysine interact in the S1’ subpocket. The region extending to the surface of the protein contains the accessory, hydrophobic S1 pocket, and an arene-binding site, where interactions with non-polar and aromatic residues in the linker region led to an increase in the binding affinity.

Other promising strategies have also been investigated for structural optimization, such as addition of an albumin-binding domain to increase the tumor uptake [35,36] or a cleavable linker moiety [37] to decrease renal toxicity. Another interesting approach is the development of multimeric or multivalent agents, containing more than one targeting moiety, which allows them to bind to their target with higher avidity and affinity. This has led to increased and prolonged tumor uptake while retaining favorable tumor to background ratios, warranting further investigation [38,39,40,41].

Herein, we designed a small library of PSMA-targeting molecules based on the glutamate-urea-lysine binding vector (EuK) and with varying linker composition, in order to obtain an optimized PSMA-targeting agent. The lead candidate was then (i) evaluated for its radiochemical and biological properties after addition of a radionuclide chelator; (ii) used as a base monomeric unit for the construction of a homobivalent ligand; (iii) radiolabeled with the diagnostic radionuclide indium-111 and tested the monomeric and dimeric agents in a PSMA-positive LS174T tumor xenograft model by in vivo SPECT/CT imaging and ex vivo biodistribution. 

## 2. Results

**Scheme 1:**
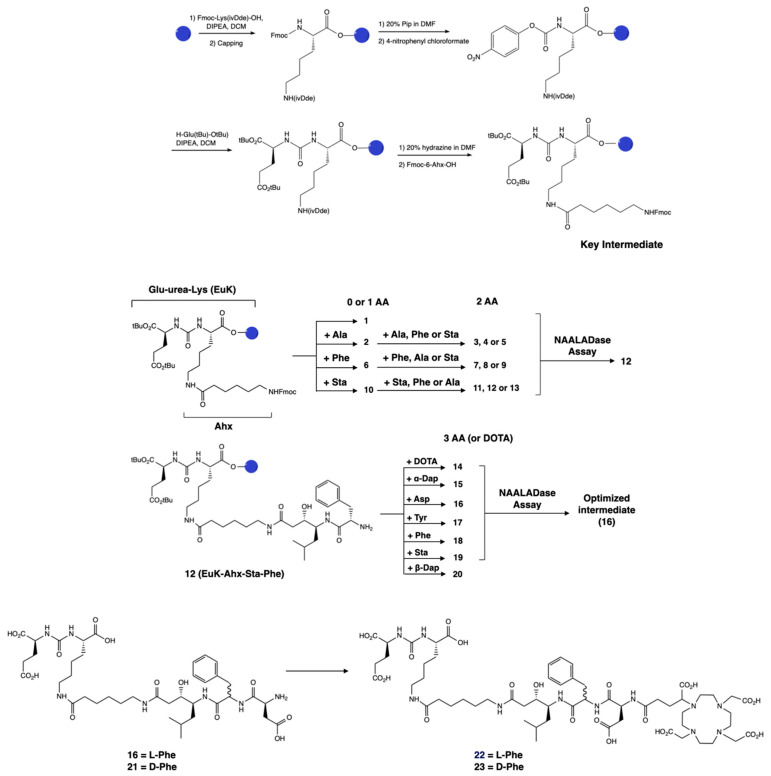
Design and synthesis of the library of PSMA-targeting compounds **1–23**. The lead candidate, **12**, was selected via a NAALADase assay from a library of 13 compounds derived from the Glu–urea–Lys moiety with the addition of 0–2 amino acids. The synthesis of the Glu–urea–Lys moiety is also shown. Then, a third amino acid was attached to the lead and these compounds were evaluated. The best candidate **16** was used as the monomeric unit for the synthesis of a dimerized compound. A DOTA-GA chelator was attached to **16** in order to evaluate its radiochemical stability, leading to **22**. The D-Phe analogs **21** and **23** were also synthesized as instability due to the presence of L-Phe has been previously reported.

The PSMA-binding moiety Glu–urea–Lys (EuK) was synthesized in solid phase by adapting a protocol described by Derks et al. [42]. The urea moiety was formed via attachment of a *p*-nitrophenyl chloroformate moiety to the protected lysine, followed by substitution with glutamic acid di-*tert*-butyl ester to yield the protected EuK moiety. This method offered a milder alternative than the use of the toxic triphosgene [33,43] and allowed for a facile solid-phase synthesis. We employed the 2-chlorotrityl chloride resin in place of the Wang resin and the 1-(4,4-dimethyl-2,6-dioxocyclohex-1-ylidene)isovaleryl (ivDde) protecting group instead of the 4-methyltrityl (Mtt) group, as the peptide was easily cleaved from the Wang resin under the acidic conditions used for Mtt deprotection. IvDde deprotection enabled the elongation of the peptide on the side chain of the lysine residue by coupling Fmoc–Ahx–OH. This intermediate was used for the preparation of compounds **1–13** (Scheme 1). Compounds **1–13** were obtained in yields of 4.5–49%. In particular, compounds **11–13**, featuring the Ahx–Ala–Sta sequence, were all obtained in low yields (4.5–7.5%). After cleavage from the resin and ether precipitation, the presence of the compounds was observed in the ether phase, which could be due to their lipophilicity. Similar phenomena could explain the low yield obtained for **2** (EuK(Ahx-Ala), 8%) despite the partial recovery of the product from the ether phase. 

The IC_50_ values of **2–13** were determined via a NAALADase assay in a single plate to avoid inter-assay variations and with PSMA-617 as a reference. All compounds exhibited binding affinity to PSMA in the nanomolar range (IC_50_ values ranging from 1.01 to 23.1 nM; Table S1), indicating that the modifications did not lead to a significant loss of affinity. The IC_50_ value of **12** (EuK(Ahx-Sta-Phe)) was comparable to the IC_50_ value measured for PSMA-617 (1.01 ± 0.4 nM for **12** vs. 0.90 ± 0.3 nM for PSMA-617). A third amino acid was added to **12** in order to obtain an appropriate length between the EuK binding motif and the chelator, but also to investigate if additional interactions could occur with the hydrophobic pocket of PSMA. We also evaluated the effect of the chelator by attaching a DOTA chelator (1,4,7,10-tetraazacyclododecane-1,4,7,10-tetraacetic acid) to **12**, generating compound **14**. Compound **14** had an IC_50_ value of 1.73 ± 0.7 nM; therefore, the presence of the chelator did not considerably affect the binding. Addition of a third amino acid containing a hydrophobic or charged residue (Asp, Sta, Phe, Tyr, or Dap) did not lead to a loss of binding (IC_50_ values ranging between 1.03 and 3.33 nM; Table S1). Consequently, we decided to proceed with **16** (EuK(Ahx-Sta-Phe-Asp), IC_50_ = 1.03 ± 0.5 nM) for further derivatization as it showed the best binding affinity from this second group of compounds. Attachment of a DOTA-GA chelator (1,4,7,10-tetraazacyclododececane,1-(glutaric acid)-4,7,10-triacetic acid) to **16** was performed to evaluate the radiochemical and biological properties of the candidate. DOTA-GA was selected instead of the usual DOTA chelator due to the presence of the Asp residue in the linker. We hypothesized that the proximity to the free carboxylic acid group of Asp could influence the coordination sphere of the chelator. Furthermore, we evaluated EuK(Ahx-Sta-Phe-Asp-DOTA-GA) containing a L- and D-Phe residue since previous studies showed enhanced metabolic stability of the sequence containing the D-Phe amino acid [44]. EuK(Ahx-Sta-L-Phe-Asp-DOTA-GA) **22** and EuK(Ahx-Sta-D-Phe-Asp-DOTA-GA) **23** were both obtained in good yields (15 and 37%, respectively) after cleavage from the resin and purification. Both compounds showed high radiochemical yield and purity (>95%, Table 1), excellent stability in labeling solution, PBS, and mouse serum up to 24 h (Figure S6), when radiolabeled with indium-111. The emulsifier Kolliphor was added to the labeling solution to prevent “stickiness” of the compounds. Compounds **22** and **23** were highly hydrophilic, as demonstrated by the logD values (**22**: −3.26 ± 0.05; **23**: −3.21 ± 0.07). No proteolytic degradation of [^111^In]In-**22** was observed, so we opted to proceed solely with the L-analog for further evaluation. 

**Scheme 2:**
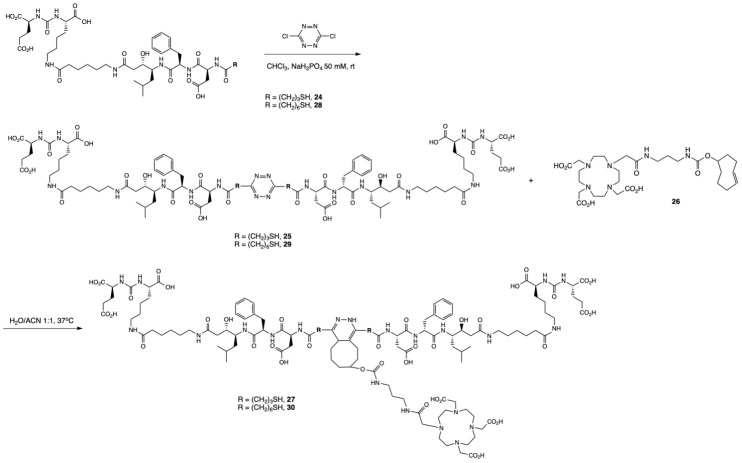
General synthetic scheme of the dimeric compounds **27** and **30**. The monomeric unit was obtained by attaching either 3-(tritylthio)propionic acid or TrtS-Hx-OH in solid phase to **16** to obtain **24** or **28**, respectively, after cleavage from the resin. Reaction of **24** with 1,2,3,5-dichloro-tetrazine provided the dimerized compound **25**, which was then clicked to the DOTA-TCO chelator (**26**) to form the final compound **27**. Similarly, the reaction of **28** with the tetrazine yielded the dimerized compound **29** and the final compound **30** after reacting with **26**. The synthesis of **24**, **25**, and **27** are detailed in the Supplementary Information.

Dimerization was performed by elongation of **16** with 3-tritylthiopropionic acid, and reaction with 3,6-dichlorotetrazine following a biphasic protocol after cleavage from the solid support [45]. Finally, addition of the chelator DOTA-TCO **26** was performed via the inverse electron-demand Diels–Alder reaction (IEDDA) to yield the dimeric product **27**. However, it was noticed that **27** was not stable in aqueous solution, with degradation observed by LC-MS 20 min after incubation in water at room temperature. The values observed in ESI-MS suggested the oxidation of **27**, which is likely occurring at one of the sulfur atoms adjacent to the tetrazine ring, followed by the release of one PSMA-binding unit. We hypothesized that it could originate from intramolecular interaction between the sulfur and the carboxylic acid group of the aspartic acid. Therefore, we modified the design of the dimer to include a longer spacer between **16** and the tetrazine. We replaced the 3-mercaptopropionic acid with a 6-mercaptohexanoic acid spacer. The resulting compound **30** (Scheme 2), obtained after dimerization and IEDDA reaction, was radiolabeled with In-111 and obtained in high RCY (>99%) and RCP (92.3%). It was stable in aqueous media and in the radiolabeling conditions, but also in PBS and mouse serum (>90% intact [^111^In]In-**30** at 24 h; Figure S7). [^111^In]In-**30** also retained a fairly hydrophilic character with a logD value of −2.44 ± 0.07, but showed increased hydrophobicity compared to [^111^In]In-**22**, likely due to the presence of the pyridazine adduct. Radiochemical data of [^111^In]In-**22** and [^111^In]In-**30** are summarized in Table 1.

**Figure 1:**
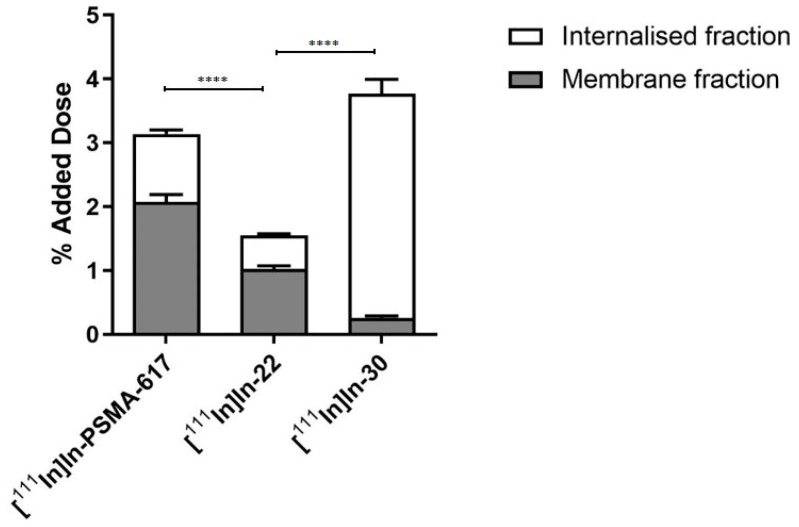
Uptake and internalization of [^111^In]In-PSMA-617, [^111^In]In-**22** and [^111^In]In-**30** in LS174T PSMA + cells. Values are expressed as % of the added dose (% AD). *p*-values smaller than *p* < 0.0001 were indicated with four (****) asterisks.

Subsequently, **22** and **30** were tested for their binding affinity towards PSMA via the NAALADase assay. Compared to the reference PSMA-617 (IC_50_ = 0.90 ± 0.30 nM), both **22** and **30** retained a high binding affinity to PSMA (**22**: IC_50_ = 1.66 ± 0.63 nM and **30**: IC_50_ = 1.05 nM ± 0.30 nM). The better affinity of **30** was expected since dimeric conjugates have been shown to display higher affinity towards their cognate receptor [40]. However, the IC_50_ values of these three compounds were not statistically different. Next, we evaluated the uptake and internalization of [^111^In]In-**22** and [^111^In]In-**30** in PSMA-overexpressing LS174T cells (Figure 1). [^111^In]In-**22** showed PSMA-specific uptake (1.55 ± 0.21% AD), which was mainly membrane bound (66%). The uptake of the monomer **22** was significantly lower than the uptake of [^111^In]In-PSMA-617 (3.14 ± 0.47% AD, 66% membrane-bound). However, [^111^In]In-**30** showed the highest PSMA-specific uptake of 3.79 ± 0.74% AD (*p* = 0.05), of which 92%, surprisingly, corresponded to the internalized fraction. For all three compounds, no uptake was observed when blocked with an excess (50-fold) of non-labelled PSMA-617. 

**Figure 2:**
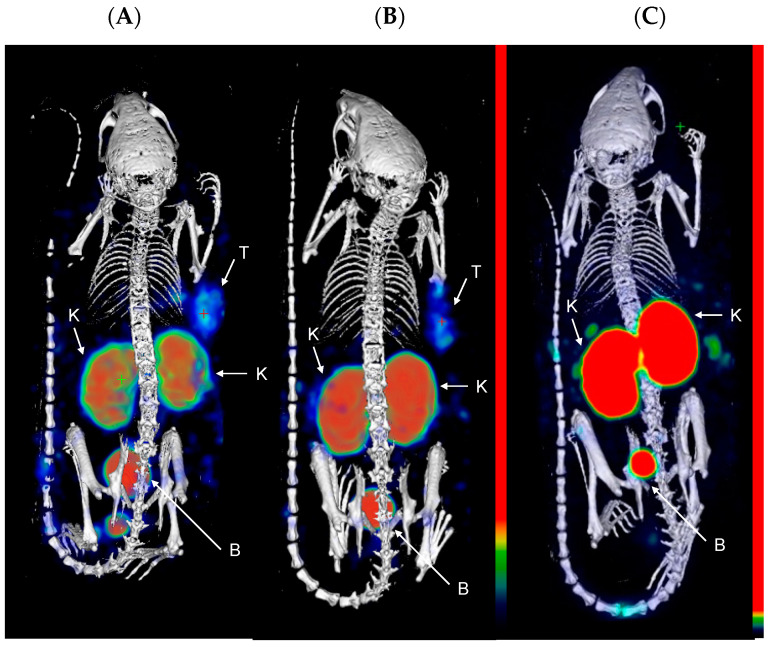
Representative MIP images of SPECT/CT scans at 1 h p.i. of (**A**) [^111^In]In-PSMA-617; (**B**) [^111^In]In-**22**; and (**C**) [^111^In]In-**30** in LS174T xenograft-bearing mice (right flank; n = 4). Color scale represents % ID/mL. The tumor (T), kidneys (K), and bladder (B) are pointed out with a white arrow.

**Figure 3:**
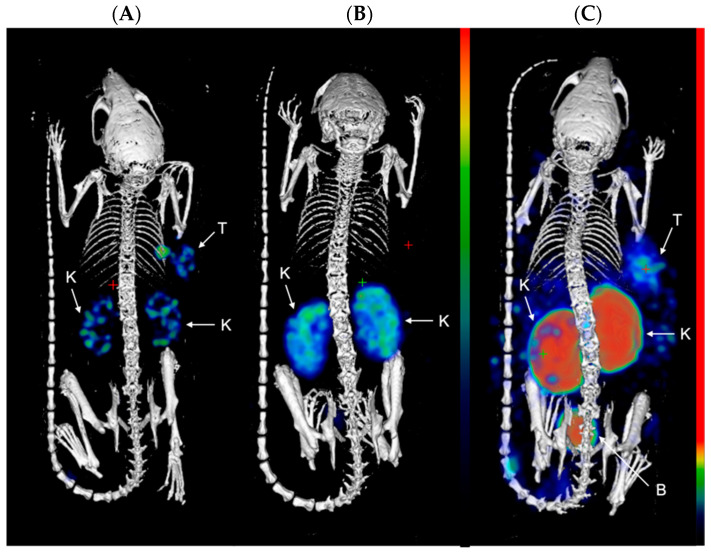
Representative MIP images of SPECT/CT scans at 24 h p.i. of (**A**) [^111^In]In-PSMA-617; (**B**) [^111^In]In-**22**; and (**C**) [^111^In]In-**30**, in LS174T xenograft-bearing mice (right flank; n = 4). Color scale represents % ID/mL. The tumor (T), kidneys (K), and bladder (B) are pointed out with a white arrow.

Next, the compounds were evaluated in vivo in BalB/C nu/nu mice bearing LS174T PSMA-positive tumor xenografts. SPECT/CT scans were performed to evaluate the biodistribution of the three compounds in vivo. Mice were injected with [^111^In]In-PSMA-617 (20.71 ± 0.53 MBq, 1.03 nmol), [^111^In]In-**22** (25.03 ± 0.59 MBq, 1.25 nmol), or [^111^In]In-**30** (26.27 ± 0.39 MBq, 1.31 nmol) and imaged at 1 and 24 h p.i. At 1 h p.i., the tumors (right flank) were clearly visible in the mice administered with [^111^In]In-PSMA-617 and [^111^In]In-**22** (Figure 2). Elevated kidney and bladder uptake were also visible due not only to the excretion route of these radiolabeled agents but also the natural expression of PSMA in the kidneys. Due to the extremely high kidney uptake of [^111^In]In-**30** at early time-points, only the kidneys and the bladder could be seen on the SPECT images at 1 h p.i.. However, at 24 h p.i., the tumor of the mice injected with [^111^In]In-**30** was clearly visualized, while tumor uptake was faint with [^111^In]In-PSMA-617 and not observable with [^111^In]In-**22** (Figure 3). These results indicate that [^111^In]In-**30** could be a suitable imaging probe at later time-points.

**Figure 4:**
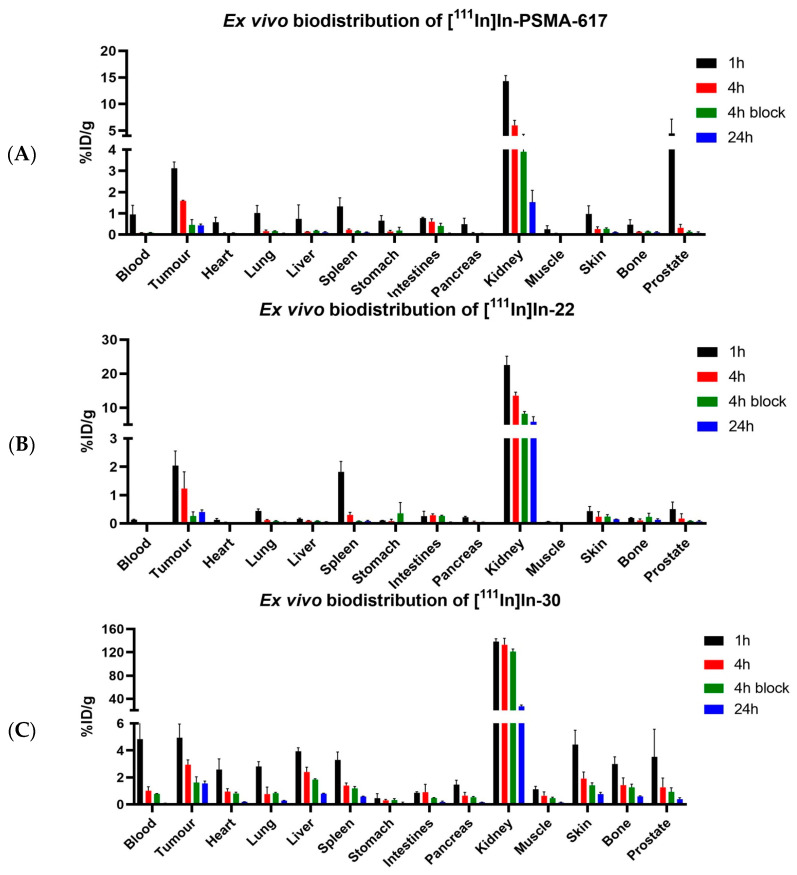
Ex vivo biodistribution of (**A**) [^111^In]In-PSMA-617, (**B**) [^111^In]In-**22** and (**C**) [^111^In]In-**30**. Organ uptake is shown as percentage of injected dose per gram. Organs were collected at 1, 4, and 24 h p.i., with a block group at 4 h p.i.

Mice were intravenously injected with [^111^In]In-PSMA-617 (5.09 ± 0.43 MBq, 1.02 nmol), [^111^In]In-**22** (6.34 ± 0.19 MBq, 1.26 nmol), or [^111^In]In-**30** (6.19 ± 0.35 MBq, 1.24 nmol), for ex vivo biodistribution studies. The results are shown in Figure 4. The values of percentage of injected dose per gram of tissue (% ID/g) for all organs can be found in Tables S4–S6. [^111^In]In-**22** showed tumor uptake of 2.05 ± 0.52% ID/g at 1 h p.i., which steadily decreased to 0.61 ± 0.40% ID/g at 24 h p.i. Compared to [^111^In]In-PSMA-617, the uptake was lower at 1 h p.i. (3.12 ± 0.30% ID/g, *p* = 0.05), but comparable at 4 h and 24 h p.i. (*p* = 0.05). In comparison, [^111^In]In-**30** showed a significantly higher uptake of 4.95 ± 1.01% ID/g at 1 h p.i. (*p* = 0.01), which was retained at 24 h p.i. (1.57 ± 0.17% ID/g). A high uptake of 4.84 ± 1.44% ID/g in the blood at 1 h p.i. indicates that this compound circulated longer in the blood. It could explain the longer retention of [^111^In]In-**30** in the tumor, but also the high background found in most organs at 1 h p.i. (liver: 3,94 ± 0.26% ID/g; skin: 4.44 ± 1.06% ID/g; lung: 2.81 ± 0.36% ID/g; bone: 3.00 ± 0.53% ID/g). Additionally, uptake was observed at 1 h p.i. for all compounds in the liver, spleen, and in the skin, as PSMA is also expressed in the endothelium [46]. The kidney uptake of [^111^In]In-**30** increased significantly compared to [^111^In]In-**22** (138.54 ± 1.44% ID/g at 1 h p.i. and 132.89 ± 11.12% ID/g at 4 h p.i., for [^111^In]In-**30**, compared to 22.56 ± 2.59% ID/g at 1 h p.i. and 13.55 ± 1.04% ID/g at 4 h p.i. for [^111^In]In-**22** (*p* < 0.001)) but decreased considerably at 24 h p.i. to 26.86 ± 2.37% ID/g for [^111^In]In-**30** and to 5.89 ± 1.49% ID/g for [^111^In]In-**22**. Co-injection of an excess of unlabeled PSMA-targeting ligand significantly reduced the tumor uptake for all compounds, proving uptake selectivity ([^111^In]In-PSMA-617 at 4 h p.i. 1.59 ± 0.03% ID/g vs. blocked 0.46 ± 0.26% ID/g (*p* < 0.01); [^111^In]In-**22** at 4 h p.i. 1.23 ± 0.59% ID/g vs. blocked 0.27 ± 0.14% ID/g (*p* = 0.01); [^111^In]In-**30** at 4 h p.i. 2.94 ± 0.36% ID/g vs. blocked 1.42 ± 0.14% ID/g (*p* < 0.01)).

**Figure 5:**
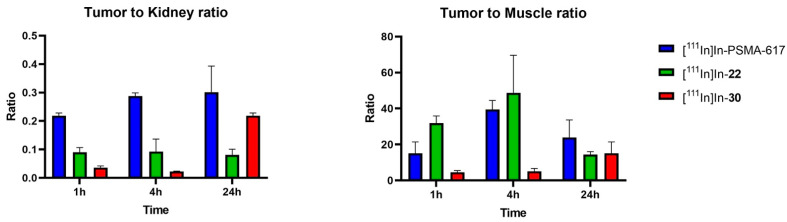
Tumor-to-kidney (T/K) and tumor-to-muscle (T/M) ratios for [^111^In]In-PSMA-617, [^111^In]In-**22** and [^111^In]In-**30** at 1, 4 and 24 h p.i. T/M ratios are used to represent tumor-to-background ratios.

The tumor-to-kidney (T/K) ratios (Figure 5) remained constant for [^111^In]In-PSMA-617 (0.2 to 0.3) and [^111^In]In-**22** (0.08 to 0.09) but increased over time for [^111^In]In-**30** (0.02 to 0.2) due to prolonged tumor retention and clearance from the kidneys at 24 h. The tumor-to-muscle (T/M) ratios were determined to evaluate the tumor-to-background levels. This ratio increased from 1 to 4 h for both [^111^In]In-PSMA-617 (15 to 39) and [^111^In]In-**22** (31 to 48) and decreased at 24 h (PSMA-617: 26; [^111^In]In-**22**: 14), suggesting that 4 h would be an ideal imaging time-point for these compounds. The T/M ratio for [^111^In]In-**30** was low at 1 and 4 h (4–5) but increased at 24 h (15), suggesting that imaging at a late time-point would be preferable with [^111^In]In-**30**). 

## 3. Discussion

Linker modifications can significantly influence the binding affinity, internalization, pharmacokinetics, and biodistribution of PSMA-targeting ligands [27]. This is mainly due to interactions between the chemical groups present in the linker and the binding pocket of PSMA. Characterization of the binding domain of PSMA led to the discovery of a S1 hydrophobic pocket, as well as an arene-binding site [47]. As expected, it was found that the binding properties of PSMA-targeting compounds were improved by increasing the lipophilicity of the linker. Compounds containing aromatic moieties in the linker region also presented high binding to PSMA [34]. We initially evaluated the effect of three amino acids (alanine (Ala), phenylalanine (Phe), and statine (Sta)) attached to the glutamate-ureido–lysine–Ahx in a combinatorial fashion (0 to 2 amino acids). The aminohexanoyl (Ahx) moiety is known to enhance the lipophilicity while allowing for flexibility and to serve as a spacer to avoid steric hindrance in the narrow binding region [34]. Ala and Phe contain hydrophobic side chains, and the presence of Phe residues has been shown to improve binding [48]. However, hydrophobic groups may influence the logD value of the compounds, and the change in lipophilicity can affect the clearance by the kidneys, as well as the background activity. We therefore also included in our investigations Sta, a non-natural amino acid containing both a hydrophilic hydroxyl group and a hydrophobic isopropyl group to balance the polarity of our linker. Extensive studies were performed by Benešová et al. [27] on the design of the linker. An optimal number of three aromatic moieties in the linker was found to improve binding affinity. However, we did not observe significant difference in the binding affinity of compounds containing one (**4**, **6**, **8**, **9**, **12**) or two (**7**) Phe residues. Biphenyl and multiring substituents have also been evaluated in different studies. While Benešová et al. [27] found that a biphenyl-residue containing derivative showed the highest *Ki*, Wirtz et al. [49] reported that a biphenylalanine-containing derivative showed a lower binding affinity, possibly due to steric repulsion in the arene-binding site. To minimize these effects, we have chosen to restrict our investigations to single aromatic rings. 

Our lead candidate, **16** (EuK(Ahx–Sta–Phe–Asp)), contains an aspartic acid residue, which brings a negative charge to the linker. This may be favorable, as charges in the linker region have also been shown to improve biodistribution of PSMA-targeting agents and lead to compounds with reduced background compared to non-charged linkers [50,51]. In our study, we compared both the sequences containing a L-Phe (**22**) and D-Phe (**23**), as proteolytic cleavage has been reported when the FFK (Phe-Phe-Lys) linker was used [44]. Both compounds [^111^In]In-**22** and [^111^In]In-**23** were obtained in high radiochemical purity (>95%) and were >90% stable in PBS and mouse serum for up to 24 h, demonstrating that the presence of L–Phe did not result into degradation of our linker. Interestingly, the Phe–Lys (FK) bond has been shown to be cleaved by enzymes present in the renal brush border membrane of the kidneys, such as the neutral endopeptidase [52], which could explain the degradation observed by the authors. The absence of a Lys would then explain why there was no loss of stability in **22**. Slight changes in the linker, including the stereochemistry of the amino acid, may also alter the properties of the final compound. In our case, while both **22** (L-Phe) and **23** (D-Phe) retained a good IC_50_, **22** had a two-fold better value than **23**.

The promising preliminary evaluation of **22** led us to consider further modifications which could improve its biological properties, such as binding affinity, uptake, and internalization, and possibly in vivo biodistribution. An effective strategy to improve binding is the attachment of an additional targeting moiety to the conjugate, creating a homobivalent or dimeric ligand. This strategy has been shown to increase tumor uptake and retention of radiopharmaceuticals, leading to higher imaging contrast [39,40]. This is due to the increased avidity and affinity of these ligands, as they present multiple copies of the pharmacophore. We employed a modular strategy relying on click chemistry, where compound **16** was dimerized by a stapling approach with 1,3-dichloro tetrazine [45]. This strategy has the advantage of a rapid formation of the dimer, and the tetrazine group can then be used to perform an IEDDA click reaction for further derivatization. A *trans*-cyclooctene functionalized DOTA chelator (**26**) was synthesized and then attached to the dimer via the click reaction [53]. The resulting compound **30** showed high affinity to PSMA and, surprisingly, a high rate of internalization. Internalization is usually desired as it favors tumor retention and increases the exposure of the tumor cells to the radiolabeled agent. Wüstemann et al. [54] evaluated the influence of different radionuclide chelators conjugated to a PSMA-binding ligand. While variations in the binding affinity were minimal, internalization rates varied from 15% to 65% in function of the chelator used (15.5 ± 7.5% for their DOTA-containing conjugate compared to 48.5 ± 16.4% for their NODAGA-conjugate and 65.4 ± 5.7% for the CHX-A’’-DTPA conjugate). They correlated the higher internalization to the increasing hydrophobicity of the chelator, indicating that interaction between the chelator and the receptor takes place during internalization. While the DOTA and CHX-A’’-DTPA compound ultimately showed comparable tumor uptake and tumor-to-blood ratios, tumor retention as well as kidney retention were prolonged for the CHX-A’’-DTPA conjugate. An increase in cell uptake and internalization in compounds with higher lipophilicity has also been reported by Wirtz et al. [49]. It can explain the very high internalization (92%) observed with [^111^In]In-**30**, where the DOTA chelator is attached to the hydrophobic pyridazine adduct, suggesting that this group also interacts with the receptor.

The design and evaluation of homobivalent agents targeting PSMA has been previously reported in the literature [39,40,55,56,57]. Banerjee et al. [39] obtained promising results with their dimerized compound [^111^In]**3**, consisting of a EuK binding moiety and a dialkyne lysine residue to incorporate the second binding unit via click chemistry. The DOTA chelator was attached to the ε-position of an additional lysine residue. [^111^In]**3** showed high tumor-to-background ratios due to prolonged retention in tumors and fast renal clearance, while the monomeric [^111^In]**5** had lower renal retention and lower tumor uptake at 24 h p.i. (23.17 ± 3.53% ID/g, compared to 34.03 ± 7.53% ID/g). Notably, [^111^In]**3** provided excellent tumor-to-background ratios at 24 h p.i., and even at 192 h p.i.. Similarly, we have obtained good tumor-to-background ratios for [^111^In]In-**30** at 24 h p.i., suggesting that further evaluation at later time-points could be interesting. Other dimeric compounds containing the EuK(Ahx) moiety have shown increased affinity and cellular uptake compared to their monomeric units, with improved tumor-to-background ratios [40,55]. In a recent proof-of-concept study by Reissig et al., a monomeric and a dimeric compound, [^225^Ac]Ac-mcp-M-PSMA and [^225^Ac]Ac-mcp-D-PSMA, respectively, were evaluated in biodistribution studies. The compounds showed high activity in the kidneys at 1 h p.i. (67.84 ± 23.92% ID/g vs. 103.03 ± 24.68% ID/g for the monomer and dimer, respectively), but this was cleared rapidly as shown by a steep decrease at 24 h p.i. (1.35 ± 0.26% ID/g vs. 7.17 ± 1.96% ID/g). The elevated kidney uptake with rapid clearance after 24 h is similar to what we observed in the biodistribution of [^111^In]In-**30** (138.54 ± 1.44% ID/g 1 h p.i., 26.86 ± 2.37% ID/g at 24 h p.i.). The monomer showed lower accumulation (6.78 ± 0.45% ID/g) compared to the dimer (12.21 ± 4.31% ID/g) at 24 h p.i., further supporting the evidence that dimeric compounds show prolonged retention in the tumor [56]. These results further illustrate the higher tumor retention of dimeric compounds compared to monomeric compounds, which we also observed when comparing [^111^In]In-**30** to compound [^111^In]In-**22**. 

Elevated kidney uptake is a persistent problem in PSMA-targeting radiopharmaceuticals, which is exacerbated by using dimeric constructs. Different strategies have been proposed to reduce renal uptake, such as co-administration of blocking agents, in vivo pretargeting, or even simply modifications to the PSMA-targeting moiety. PMPA (2-(phosphonomethyl)pentanedioic acid), a PSMA inhibitor, has been investigated preclinically and showed to reduce renal uptake in doses as low as 0.2 mg/kg [58]. Another strategy to reduce the kidney uptake of [^177^Lu]Lu-PSMA is co-administration with an excess of non-radiolabeled PSMA-targeting compound such as PSMA-11 [59]. Despite a 44.5% decrease in tumor-uptake due to the reduction of the effective molar activity (as a consequence of the co-administration of a maximum dose of 2000 pmoles PSMA-11), kidney and salivary gland uptake decreased by 99.5% and 89.5%, respectively, demonstrating the potential of this strategy. Co-administration of monosodium glutamate with [^68^Ga]-PSMA-11 was also shown to reduce the kidney and salivary gland uptake in mice bearing LNCaP tumor xenografts. Monosodium glutamate is a weak inhibitor of PSMA (*Ki* = 0.90 µM), suggesting that the uptake in these organs is mediated by the glutamate receptor [60]. Co-administration of [^111^In]In-**30** with one of these agents could therefore be used as a strategy to reduce its kidney uptake.

Another possibility to reduce kidney uptake would be to use an in vivo pretargeting approach, which would be possible due to the click chemistry used on our construct. This approach is based on the separate administration of the tumor-binding agent, which first accumulates in the tumor site, followed by the administration of the radiolabeled probe to form the compound in situ. This could be adapted to our compound by administering the radiolabeled [^111^In]In-DOTA-TCO at a later time point and then the dimerized **29**, i.e., when the T/K ratio would be optimal. This approach has shown promise with the clinical translation of a biopolymeric system conjugated to a protodrug of Doxorubicin [61] and with larger conjugates such as the diabody AVP04-07 for imaging radioimmunotherapy [62]. However, it still needs to be further validated for low-molecular-weight radiopharmaceuticals [63]. 

Perhaps the key to reducing kidney uptake lies not only in the optimization of the linker moiety but also the targeting vector. Kuo [64] et al. have recently reported that replacing the glutamate moiety of the PSMA-targeting ligand with Asp, Aad (L-2-aminoadipic acid), and Api (2-aminopimelic acid) led to improvements in tumor-to-kidney and tumor-to-salivary gland ratios in vivo. However, Felber et al. [65] also recently evaluated a series of PSMA-targeting analogs with modifications within the urea group of a EuE binding unit, using a thioureate and carbamate derivatives. They also evaluated proinhibitors and modifications within the γ-carboxylic acid of the glutamate residue. With the exception of a carbamate and tetrazole derivative which retained nanomolar-range binding affinities, they observed loss of affinity for all other modifications, illustrating that modifications in the binding moiety must be proceeded with caution. Nevertheless, replacing the PSMA-targeting EuK moiety of **30** by one of these alternative sequences could potentially lead to a decrease of the kidney uptake and improved tumor-to-background ratios. Compound **30** showed promising characteristics for RLT, such as extended circulation time and high tumor accumulation and retention. Therefore, the aforementioned approaches would be particularly attractive to decrease the off-target toxicity of **30**, when it will be associated to an α- or β- emitter.

Finally, a limitation of this study is that we did not compare the uptake and biodistribution of [^111^In]In-**30** with its true monomeric unit, consisting of a single EuK(Ahx-Sta-Phe-Asp-Shx) sequence attached to the tetrazine and clicked to the TCO-DOTA moiety. Therefore, we cannot conclude if the differences in biodistribution observed between [^111^In]In-**22** and [^111^In]In-**30** are only due to the presence of the additional binding moiety or if it partially comes from the modification in the linker and inclusion of the pyridazine. As previously mentioned, charges in the linker region have been shown to reduce off-target retention. [^111^In]In-**22** possesses an additional charge in the linker region due to the presence of the Asp residue (on top of the charges from the three carboxylic acid residues from the EuK binding vector) and showed very low off-target retention (with the exception of some spleen uptake at 1 h p.i., which is rapidly washed out). [^111^In]In-**30**, possessing two targeting vectors and therefore additional charges, however, showed higher uptake in non-targeted organs. We also observed that while our latest time-point of 24 h was sufficient for the imaging and biodistribution of [^111^In]In-PSMA-617 and [^111^In]In-**22**, later time-points are required to better characterization of the pharmacokinetics of [^111^In]In-**30**. The promising results reported thus far warrant further evaluation of these dimeric constructs at later time-points and at different dosages, but also as potential theranostic pairs in therapy studies.

## 4. Materials and Methods

### 4.1. General Methods

All chemicals and solvents were obtained from commercial suppliers in reagent grade or better and were used without further purification unless specified. [^111^In]InCl_3_ was purchased from Curium (Petten, The Netherlands). Solid phase reactions were performed using a SiliCycle MiniBlock system (Quebec, QB, Canada) [66] or using manual peptide synthesis vessels (Chemglass; Vineland, NJ, USA). Reactions were monitored using the Kaiser and/or TNBS test. Incomplete couplings and deprotections were repeated. PSMA-617 was synthesized as a reference according to the literature [43]. Instant thin-layer iTLC-SG chromatography plates (Agilent; Folsom, CA, USA) were analyzed by a bSCAN scanner (Brightspec; Antwerp, Belgium). Radioactivity in biological experiments was measured using a PerkinElmer Wizard 2 gamma counter (Groningen, The Netherlands). 

#### High Performance Liquid Chromatography (HPLC) Conditions

Reactions were monitored by liquid chromatography–mass spectrometry (LC-MS) using an Agilent 1260 Infinity II LC/MSD XT system (Amstelveen, The Netherlands) and an Agilent Infinity Lab Poroshell 120 EC-C18 column (3 × 100 mm, 2.7 µm). The mobile phase consisted of the following: Solvent A, 0.1% formic acid (FA) in water; Solvent B, 0.1% FA in acetonitrile (ACN). The following LC gradient was used for all analyses: 0–5 min; 5–100% B; and 5–8 min, 100% B. The flow rate was 0.5 mL/min, and the chromatograms were recorded at either 220 or 254 nm. Samples were diluted in H_2_O/ACN 1:1 for a final volume of 5–10 µL. 

Purifications were performed on a preparative HPLC system (Agilent 1290 Infinity II) using an Agilent 5 Prep C18 column (50 × 21.2 mm, 5 µm). The mobile phase consisted of the following: Solvent A, 0.1% FA in water; Solvent B, 0.1% FA in ACN. The default gradient used was 0–8 min, 5–100% B, and 8–10 min, 100% B. The flow rate was 10 mL/min, and chromatograms were recorded at either 220 or 254 nm using the OpenLab CDS Chemstation software (Agilent). Samples were diluted in H_2_O/ACN 1:1 and injection volumes ranged from 50 to 200 µL.

Radio-HPLC was performed with a Waters Alliance e2695 system (Etten-Leur, The Netherlands) equipped with a 2998 diode array (PDA) detector for UV detection; a NaI(Tl) Scionix crystal (Bunnik, The Netherlands) connected to a Canberra Osprey multichannel analyzer; and a signal amplifier (Zellik, Belgium) for detection of the radioactive signal. Empower 3 software was used to analyze the chromatograms. The reversed-phase analytical Gemini C18 column (250 × 4.6 mm, 5 µm) from Phenomenex (Torrance, CA, USA) was used for all analyses. The mobile phase consisted of the following: Solvent A, 0.1% trifluoroacetic acid (TFA) in H_2_O; Solvent B, 0.1% TFA in ACN. Analysis was performed at a flow rate of 1 mL/min using the following gradient of solvents A and B: 0–3 min, 5% B; 3–23 min, 5–100% B; 23–27 min, 100% B. HPLC eluates were monitored for their UV absorbance at 254 nm. Samples were diluted in H_2_O/ACN 1:1 and injection volumes were of 50 µL. 

### 4.2. Chemistry

#### 4.2.1. Synthesis of a Library of PSMA-Targeted Ligands

2-chlorotrityl chloride resin (938 mg, 1.5 mmol,) was swollen in dichloromethane (DCM; 10 mL) for 30 min. A solution of Fmoc-L-Lys(ivDde)-OH (3.49 g, 6 mmol, 4 equiv.) in DCM (10 mL) and *N*,*N*-diisopropylethylamine (DIPEA; 870 μL, 7.5 mmol, 5 equiv.) was added to the resin, and the mixture was agitated for 2 h. The resin was washed with dimethylformamide (DMF; 3 × 10 mL), ethanol (EtOH; 2 × 10 mL), and diethyl ether (2 × 10 mL) and dried. The Fmoc-loading was determined to be 0.74 mmol/g [67]. The resin was then capped with DCM/MeOH/DIPEA (10 mL; *v*/*v*/*v*, 80:15:5) for 30 min. The Fmoc group was deprotected (5 + 10 min) with a solution of 4-methylpiperidine in DMF (10 mL; *v*/*v*, 1:4), and the resin was washed with DMF (5 × 10 mL). A solution of 4-nitrophenyl chloroformate (282 mg, 1.4 mmol, 2 equiv., resin loading 0.74 mmol/g) and DIPEA (365 µL, 2.1 mmol, 3 equiv.) in 5 mL of DCM was added to the resin and agitated for 1 h. The solution was filtered, and reaction completion was checked by Kaiser test. Then, a solution of glutamic acid di-*tert*-butyl ester hydrochloride (621 mg, 2.1 mmol, 3 equiv.) and DIPEA (487 µL, 2.8 mmol, 4 equiv.) in 5 mL of DCM was added to the resin and agitated for 1 h. Reaction completion was monitored by LC-MS analysis of a sample cleaved from the solid support, using 2 mL of a TFA/TIS/H_2_O (*v*:*v*:*v* 95:2.5:2.5) cocktail. The resin was then washed with DMF (5 × 10 mL) [42]. The ivDde group was deprotected by treatment (2 × 15 min) of the resin with a solution of 2% hydrazine in DMF (10 mL), and the resin was washed with DMF (5 × 10 mL). A solution of Fmoc–Ahx–OH (989 mg, 2.8 mmol, 4 equiv.) in 10 mL DMF with HBTU (1.04 g, 2.7 mmol, 3.9 equiv.), Oxyma Pure (398 mg, 2.8 mmol, 4 equiv.), and DIPEA (1.22 mL, 7 mmol, 10 equiv.) was added to the resin and agitated for 45 min. The resin was washed with DMF (5 × 10 mL) and the Fmoc group was deprotected as previously described to obtain *t*Bu–O–Glu(*t*Bu)–urea–Lys(Ahx) resin, which was the key intermediate for the synthesis of compound **1–13** (Scheme 1). Further elongation of 12 with an additional amino acid or DOTA chelator gave compounds **14–20** (Figure S1). Further details about the synthesis and characterization of each compound can be found in the Supplementary Information.

#### 4.2.2. Synthesis of EuK(Ahx-Sta-L-Phe-Asp-DOTA-GA) (**22**)

To **16** (0.05 mmol) was added a solution of DOTA-GA(tBu)_4_ (70 mg, 0.10 mmol, 2 equiv.) in 4 mL of DMF with benzotriazol-1-yloxytripyrrolidinophosphonium hexafluorophosphate (PyBOP; 52 mg, 0.10 mmol, 2 equiv.) and DIPEA (35 µL, 0.20 mmol, 4 equiv.). The resin was agitated overnight at room temperature. When the reaction was complete, the beads were washed with DMF (5 × 10 mL). The product was cleaved from the resin using a solution of TFA/TIS/H_2_O (*v*:*v*:*v* 95:2.5:2.5) for 2 h, rt, and the compound was precipitated with ice-cold diethyl ether. Additional deprotection step was performed by dissolving the crude product in 1 mL of a TFA/H_2_O/TIPS mixture and monitoring by LC-MS. HPLC purification provided 22 as a white solid (9.6 mg, 7.3 μmol, 15%), which was characterized by LC-MS (t_R_ = 3.62 min, purity > 95%). ESI-MS *m/z*: calc’d. for C_58_H_91_N_11_O_23_: 1309.63; found: 1310.60 [M+H]^+^. 

#### 4.2.3. Synthesis of (E)-2,2′,2″-(10-(2-((3-(((cyclooct-4-en-1-yloxy)carbonyl)amino)propyl)amino)-2-oxoethyl)-1,4,7,10-tetraaza cyclododecane-1,4,7-triyl)triacetic acid, DOTA-TCO (**26**)

DOTA-NHS ester (12.7 mg, 16.7 mmol, 1.4 equiv.) and TCO-amine HCL salt (3.2 mg, 12.1 μmol, 1 equiv.) were solubilized in DMF (200 μL), to which DIPEA (13.5 μL, 77 μmol, 6.5 equiv.) was added. The reaction was stirred at room temperature and monitored by LC-MS. After 2 h, full consumption of the TCO-amine indicated reaction completion. The solvent was removed under vacuum, and the crude was redissolved in H_2_O/ACN (1:1) and purified by HPLC (*t_R_* = 3.47 min). Compound **26** was obtained as a white solid (5.3 mg, 8.6 μmol, 70% yield) and was characterized by LC-MS (*t_R_* = 3.71 min, purity > 95%). ESI-MS *m/z*: calc’d for C_28_H_48_N_6_O_9_ 612.35; found 613.30 [M+H]^+^.

#### 4.2.4. Synthesis of EuK(Ahx-Sta-L-Phe-Asp-SHx) (**28**)

To **16** (0.05 mmol) in solid phase was added a solution of TrtS-Hx-OH (156 mg, 0.40 mmol, 4 equiv.) in DMF with HBTU (148 mg, 0.39 mmol, 3.9 equiv.), Oxyma Pure (57 mg, 0.40 mmol, 4 equiv.), and DIPEA (87 μL, 0.50 mmol, 10 equiv.), and the resin was agitated for 45 min. Compound **28** was obtained after cleavage from the resin and purification by preparative HPLC (*t_R_* = 4.85 min) as a white solid (19.6 mg, 19.9 μmol, 20% yield) and was characterized by LC-MS (*t_R_* = 4.61 min, purity > 95%). ESI-MS *m/z*: calc’d for C_45_H_71_N_7_O_15_S 981.47; found 982.50 [M+H]^+^.

#### 4.2.5. Synthesis of [EuK(Ahx-Sta-L-Phe-Asp-SHx)]_2_-Tz (**29**)

A total of 6.7 mg of 28 (6.8 μmol, 1 equiv.) was solubilized in 3.75 mL of a solution of 50 mM NaH_2_PO_4_ (pH 5, 2 mM concentration of peptide in solution). A solution of dichlorotetrazine (2.9 mg, 20.1 μmol, 3 equiv.) in CHCl_3_ (3.75 mL, equal volume to peptide) was added to **28** and both phases were stirred vigorously for 1 min. The mixture was centrifuged for 1 min at 2500 rpm and the aqueous phase was collected. The organic layer was extracted with an additional portion of water then centrifuged once more at 2500 rpm for 1 min. The combined water fractions were lyophilized, and the crude product was then purified by HPLC (*t_R_* = 4.61 min). Compound **29** was obtained as a pink solid (1.7 mg, 0.83 umol, 24% yield and was characterized by LC-MS (*t_R_* = 4.71 min, purity > 95%). ESI-MS *m/z*: calc’d for C_92_H_140_N_18_O_30_S_2_ 2042.35; found 2043.20 [M+H]^+^; 1021.70 [M+2H]^2+^.

#### 4.2.6. Synthesis of [EuK(Ahx-Sta-L-Phe-Asp-SHx)]_2_-Tz-TCO-DOTA (**30**)

Compound **29** (0.7 mg, 0.34 μmol, 1 equiv.) was solubilized in 400 µL of H_2_O/ACN (*v*/*v*, 1:1). A solution of 26 (21 µL, 10 mg/mL, 1 equiv.) in H_2_O/ACN (*v*/*v*, 1:1) was added and the mixture was stirred at 37 °C for 30 min. The orange-pink solution became colorless when the reaction was completed. Solvents were removed under reduced pressure and the residue was purified by HPLC to yield 30 as a white solid (0.8 mg, 0.30 μmol, 90%), which was characterized by LC-MS (*t_R_* = 4.30 min, purity > 95%). ESI-MS *m/z*: cal’d for C_120_H_188_N_22_O_39_S_2_: 2625.28; found: 1313.20 [M+2H]^2+^; 875.80 [M+3H]^3+^.

### 4.3. Radiochemistry

#### 4.3.1. Labeling with [^111^In]InCl_3_

All compounds were titrated prior to the labeling as previously described [68]. Labelings were performed in a solution containing sodium acetate (1 μL, 2.5 M), ascorbic, and gentisic acids (10 μL, 50 mM), L-methionine (10 μL, 50 mM), and Milli-Q water complemented with Kolliphor (2 mg/mL). Compound **22** or **30** (1 nmol) and [^111^In]InCl_3_ (50 MBq, 370 MBq/mL) were added, and the reaction mixture was heated at 90 ^o^C for 5 min. Quality control was performed using iTLC-SG plates eluted with a solution of sodium citrate (0.1 M, pH 5.0). Diethylenetriaminepentaacetic acid (DTPA, 5 μL, 4 mM) was added to complex free indium-111 before injection onto analytical radio-HPLC.

#### 4.3.2. Stability in Phosphate-Buffered Saline (PBS)

[^111^In]In-**22** or [^111^In]In-**30** (~10 MBq, 20 µL) was mixed with 80 μL of PBS (0.01 M, pH 7.4) and incubated at 37 °C for up to 24 h. A sample of the PBS solution was loaded directly onto the radio-HPLC at 1, 4, and 24 h post-incubation.

#### 4.3.3. Stability in Mouse Serum

[^111^In]In-**22** or [^111^In]In-**30** (~35 MBq, 70 µL) was mixed with 330 μL of mouse serum and incubated at 37 °C for up to 24 h. After 1, 4, and 24 h incubation, 50 μL of the mouse serum solution was added to a separate vial containing 50 µL of acetonitrile. The sample was centrifuged at 5000× *g* for 20 min. The supernatant was analyzed by radio-HPLC. 

#### 4.3.4. Determination of LogD_7.4_ Value

The distribution coefficients (LogD_7.4_ values) were determined by a shake-flask method, in which 2 μL (~0.5 MBq) of the labeling solution ([^111^In]In-**22** or [^111^In]In-**30**) were added to an Eppendorf tube containing 1 mL of PBS (0.01 M, pH 7.4)/n-octanol (*v*/*v*, 1:1). After vigorous vortexing, the solution was centrifuged at 2500× *g* for 15 min for phase separation. Samples (3 × 10 μL) of the two phases were taken out and measured in a gamma counter. LogD_7.4_ values were calculated by using the following equation: LogD_7.4_ = log [(counts in octanol phase)/(counts in aqueous phase)] and the experiment was performed in triplicate [69].

### 4.4. Biological Assays

#### 4.4.1. NAALADase Assay

Recombinant human PSMA (rhPSMA; R&D systems, Abingdon, UK) was diluted in assay buffer (50 mM HEPES, 0.1 M NaCl, pH 7.5) to 0.4 μg/mL. The substrate Ac–Asp–Glu (40 μM) was mixed with the synthesized derivatives and with a reference (PSMA-617) at concentrations ranging from 10^−6^ to 10^−13^ M in a final volume of 12.5 μL assay buffer. The mixtures were combined with 12.5 μL of the rhPSMA solution and incubated for 1 h at 37 °C in 384-well black polystyrene microplates (ThermoFisher, Bleiswijk, The Netherlands). The amount of glutamate released was measured by incubating with 25 μL of a working solution of the Amplex Red glutamic acid kit (ThermoFisher, Bleiswijk, The Netherlands) at 37 °C for 30 min. Fluorescence was measured with a HIDEX Sense Optical System (Goedereede, The Netherlands) with excitation at 535 nm and emission at 590 nm. Data were normalized and analyzed using a one-site total binding regression with GraphPad Prism v9.0 (GraphPad Software, San Diego, CA, USA).

#### 4.4.2. Cell Culture

LS174T human colon carcinoma cells transfected with human PSMA were obtained from Dr. Sandra Heskamp (Radboud UMC, Nijmegen). Cells were cultured in RPMI-1640 media supplemented with 2 mM glutamine, 10% fetal bovine serum (FBS) and 0.3 mg/mL G418 (Geneticin, Sigma Aldrich, St. Louis, MO, USA).

#### 4.4.3. Internalization and Cell Uptake Assay

Cells were seeded (1.2 × 10^6^ cells/well) and cultured to confluency for 24 h prior to experiments. We prepared 10^−9^ M solutions of [^111^In]In-PSMA-617, [^111^In]In-**22**, and [^111^In]In-**30** in internalization media (RPMI, 20 mM HEPES, 1% BSA, pH 7.4). The media were removed from each well, and they were rinsed twice with PBS at rt. 2 mL of the radioactive compound was then added to each well and incubated at 37 °C for 2 h. For the internalization assay, the media containing radioactive compound were removed, and each well was washed twice with cold PBS. A total of 1 mL of Glycine buffer (50 mM glycine, 100 mM NaCl, pH 2.8) was added immediately and incubated for 10 min at rt. The glycine wash was then collected into counting tubes (membrane-bound fraction). An extra wash was performed and collected in the same tubes. A total of 1 mL of NaOH (1 M) was then added to each tube and left for 15 min at rt. The lysate (internalized fraction) was then collected in separate counting tubes, together with one extra NaOH wash. Data were normalized to 1,000,000 cells and percentage added dose. 

### 4.5. In Vivo Studies

#### 4.5.1. Mouse Model

Animal experiments were performed in 8–10 weeks old male BALB/c nude mice (Janvier Labs, Le Genest-Saint-Isle, France). Animals were housed in individually ventilated cages (Blue line IVC, 4 mice per cage) under nonsterile standard conditions with cage enrichment present and free access to chlorophyll-free animal chow (Sniff GmbH, Soest, Germany) and water and acclimated for 1 week before experiments. Mice were subcutaneously inoculated with 4.0 × 10^6^ LS174T cells in the right flank, diluted in 100 μL of cell suspension. Seven days after tumor cell inoculation, when xenografts were approximately 0.2–0.3 cm^3^, the tracers were injected intravenously via the tail vein. All experiments were approved by the institutional Animal Welfare Committee and were conducted in accordance with the guidelines of the Revised Dutch Act on Animal Experimentation (WOD).

#### 4.5.2. Biodistribution Studies

Mice were intravenously injected with ~1 nmol of either [^111^In]In-PSMA-617, [^111^In]In-**22** or [^111^In]In-**30** with molar activity of 5 MBq/nmol for ex vivo biodistribution studies. Mice (n = 4/timepoint) were euthanized by cervical dislocation at 1, 4, and 24 h p.i. A block group was included, and mice were euthanized at 4 h p.i. Organs were collected in tubes and counted in the gamma counter, where the calibration factor was determined by measuring different known activities of indium-111. 

#### 4.5.3. SPECT/CT Imaging

Mice were intravenously injected with 1 nmol of [^111^In]In-PSMA-617, [^111^In]In-**22**, or [^111^In]In-**30** (n = 4 mice/timepoint) with molar activity of 20 MBq/nmol. At 1 and 24 h p.i., mice were placed on a heated bed under 2% isoflurane/O_2_ anesthesia and imaged in a dedicated small-animal PET/SPECT/CT scanner (VECTor^5^CT scanner, MILabs B.V., Utrecht, The Netherlands) with a high sensitivity pinhole collimator (XXUHS-M, 3.00 mm pinhole diameter). Whole-body SPECT images (transaxial field of view—54 mm) were acquired over 30 min using a spiral scan in normal scan mode in list-mode acquisition. This was followed by a whole-body CT scan within 5 min, with the following imaging settings: full angle scan, angle step 0.75 degrees, normal scan mode, 50 kV tube voltage, 0.21 mA tube current, 500 µm aluminum filter. Reconstruction of the SPECT images was performed using the similarity-regulated SROSEM method and MLMN method (MILabs Rec 12.00 software) performing 9 and 128 iterations, respectively, at 0.8 mm^3^ resolution, using 173 keV ± 10% and 247 keV ± 10% energy windows for In-111. Two adjacent background windows per photo peak were used for triple-energy window scatter and crosstalk correction. Reconstructed volumes of SPECT scans were post-filtered with an isotropic 3-dimensional Gaussian filter of 1 mm full width at half-maximum. The CT and registered attenuation-corrected SPECT images were analyzed using IMALYTICS Preclinical 3.0 (Gremse-IT GmbH, Aachen, Germany). 

#### 4.5.4. Statistical Analysis

Statistical analysis was performed using GraphPad Prism v9. Outliers testing was performed using the Grubbs test and normality was tested using the Shapiro–Wilk normality test. Significant differences for the competitive binding, uptake, internalization, and ex vivo biodistribution of [^111^In]In-PSMA-617, [^111^In]In-**22**, and [^111^In]In-**30** were evaluated using an unpaired t-test or a Mann–Whitney U test. The difference was considered statistically significant if the *p*-value was < 0.05. *p*-values smaller than 0.05 (*p* < 0.05), *p* < 0.01, *p* < 0.001, and *p* < 0.0001 were indicated with one (*), two (**), three (***), or four (****) asterisks, respectively. Data are reported as average ± standard deviation. 

## 5. Conclusions

Linker modifications consisting of 1–3 amino acids to a PSMA-targeting EuK(Ahx) vector were well tolerated, with the compounds retaining binding to PSMA in the nanomolar range. The EuK(Ahx–Sta–Phe–Asp) (**16**) sequence provided the best binding affinity to PSMA among the library of compounds, which is attributed to its hydrophobicity, the presence of an aromatic group, and a negative charge on the linker. Attachment of a DOTA-GA chelator for radiochemical evaluation using indium-111 yielded [^111^In]In-**22**, which showed good tumor targeting properties with low off-target retention in LS174T PSMA-positive tumor xenograft models. The homobivalent [^111^In]In-**30** was designed based on this sequence, and it demonstrated high uptake and internalization in LS174T cells. [^111^In]In-**30** showed increasing T/M and T/K ratios over time and good tumor targeting properties at 24 h p.i. in LS174T tumor xenograft models, despite initially elevated kidney uptake. Further evaluation of this compound at later time-points is required to determine whether it shows promise as a theranostic agent.

## Figures and Tables

**Table 1:** Radiochemical data obtained for [^111^In]In-**22** and [^111^In]In-**30**.

Compound	RCY (%)	RCP (HPLC, %)	LogD	Stability in PBS (%) ^1^	Stability in Mouse Serum (%) ^1^
[^111^In]In-**22**	99	97.4	−3.26 ± 0.05	95.4	95.1
[^111^In]In-**30**	99	92.3	−2.44 ± 0.07	90.8	90.1

^1^ Values refer to the timepoint of 24 h post-incubation.

## Data Availability

The data present in this study are available in the body of the manuscript and in the Supplementary Materials.

## Supplementary Materials

The following supporting information can be downloaded at: https://www.mdpi.com/article/10.3390/molecules28104022/s1, Chemical Synthesis of compounds **1–30**; Figure S1: Structure of compounds **1** to **23**; Figure S2: Characterization of compounds **1** to **30**; Table S1: Summary of the library compounds **1** to **20**; Figure S3: Radio-HPLC chromatogram of [^111^In]In-**22**; Figure S4: Radio-HPLC chromatogram of [^111^In]In-**30**; Figure S5: Radio-HPLC chromatogram of [^111^In]In-**27**; Figure S6: Stability of [^111^In]In-**22**; Figure S7: Stability of [^111^In]In-**30**; Figure S8: NAALADase assay of PSMA-617, **22** and **30**; Table S2: Numerical values of the IC_50_ obtained for PSMA-617, **22** and **30** via the NAALADase assay; Table S3: Numerical values of the uptake and internalization of [^111^In]In-PSMA-617, [^111^In]In-**22** and [^111^In]In-**30** in PSMA expressing LS174T cells; Table S4: Ex vivo biodistribution data of [^111^In]In-PSMA-617; Table S5: Ex vivo biodistribution data of [^111^In]In-**22**; Table S6: Ex vivo biodistribution data of [^111^In]In-**30**.

## Sample Availability

Samples of the compounds are not available from the authors.

## References

[1] Sung H., Ferlay J., Siegel R.L., Laversanne M., Soerjomataram I., Jemal A., Bray F. (2021). Global Cancer Statistics 2020: GLOBOCAN Estimates of Incidence and Mortality Worldwide for 36 Cancers in 185 Countries. CA Cancer J. Clin..

[2] Icten O., Kose D.A., Matissek S.J., Misurelli J.A., Elsawa S.F., Hosmane N.S., Zumreoglu-Karan B. (2018). Gadolinium Borate and Iron Oxide Bioconjugates: Nanocomposites of next Generation with Multifunctional Applications. Mater. Sci. Eng. C.

[3] Ailuno G., Balboni A., Caviglioli G., Lai F., Barbieri F., Dellacasagrande I., Florio T., Baldassari S. (2022). Boron Vehiculating Nanosystems for Neutron Capture Therapy in Cancer Treatment. Cells.

[4] Derks Y.H.W., Schilham M.G.M., Rijpkema M., Smeets E.M.M., Amatdjais-Groenen H.I.V., Kip A., van Lith S.A.M., van de Kamp J., Sedelaar J.P.M., Somford D.M. (2023). Imaging and Photodynamic Therapy of Prostate Cancer Using a Theranostic PSMA-Targeting Ligand. Eur. J. Nucl. Med. Mol. Imaging.

[5] Powers E., Karachaliou G.S., Kao C., Harrison M.R., Hoimes C.J., George D.J., Armstrong A.J., Zhang T. (2020). Novel Therapies Are Changing Treatment Paradigms in Metastatic Prostate Cancer. J. Hematol. Oncol..

[6] Sartor O., de Bono J., Chi K.N., Fizazi K., Herrmann K., Rahbar K., Tagawa S.T., Nordquist L.T., Vaishampayan N., El-Haddad G. (2021). Lutetium-177-PSMA-617 for Metastatic Castration-Resistant Prostate Cancer. N. Engl. J. Med..

[7] Hofman M.S., Emmett L., Sandhu S., Iravani A., Joshua A.M., Goh J.C., Pattison D.A., Tan T.H., Kirkwood I.D., Ng S. (2021). [^177^Lu]Lu-PSMA-617 versus Cabazitaxel in Patients with Metastatic Castration-Resistant Prostate Cancer (TheraP): A Randomised, Open-Label, Phase 2 Trial. Lancet.

[8] Emmett L., Subramaniam S., Joshua A.M., Crumbaker M., Martin A., Zhang A.Y., Rana N., Langford A., Mitchell J., Yip S. (2021). ENZA-p Trial Protocol: A Randomized Phase II Trial Using Prostate-Specific Membrane Antigen as a Therapeutic Target and Prognostic Indicator in Men with Metastatic Castration-Resistant Prostate Cancer Treated with Enzalutamide (ANZUP 1901). BJU Int..

[9] Sandhu S., Joshua A.M., Emmett L., Spain L.A., Horvath L., Crumbaker M., Anton A., Wallace R., Pasam A., Bressel M. (2022). PRINCE: Phase I Trial of 177 Lu-PSMA-617 in Combination with Pembrolizumab in Patients with Metastatic Castration-Resistant Prostate Cancer (MCRPC). J. Clin. Oncol..

[10] Suman S., Parghane R.V., Joshi A., Prabhash K., Talole S., Basu S. (2021). Combined (177) Lu-PSMA-617 PRLT and Abiraterone Acetate versus (177) Lu-PSMA-617 PRLT Monotherapy in Metastatic Castration-Resistant Prostate Cancer: An Observational Study Comparing the Response and Durability. Prostate.

[11] Dhiantravan N., Emmett L., Joshua A.M., Pattison D.A., Francis R.J., Williams S., Sandhu S., Davis I.D., Vela I., Neha N. (2021). UpFrontPSMA: A Randomized Phase 2 Study of Sequential 177Lu-PSMA-617 and Docetaxel vs Docetaxel in Metastatic Hormone-Naïve Prostate Cancer (Clinical Trial Protocol). BJU Int..

[12] Dhiantravan N., Violet J., Eapen R., Alghazo O., Scalzo M., Jackson P., Keam S.P., Mitchell C., Neeson P.J., Sandhu S. (2021). Clinical Trial Protocol for LuTectomy: A Single-Arm Study of the Dosimetry, Safety, and Potential Benefit of (177)Lu-PSMA-617 Prior to Prostatectomy. Eur. Urol. Focus.

[13] Privé B.M., Janssen M.J.R., Van Oort I.M., Muselaers C.H.J., Jonker M.A., De Groot M., Mehra N., Verzijlbergen J.F., Scheenen T.W.J., Zámecnik P. (2020). Lutetium-177-PSMA-I&T as Metastases Directed Therapy in Oligometastatic Hormone Sensitive Prostate Cancer, a Randomized Controlled Trial. BMC Cancer.

[14] Yadav M.P., Ballal S., Sahoo R.K., Dwivedi S.N., Bal C. (2019). Radioligand Therapy With 177Lu-PSMA for Metastatic Castration-Resistant Prostate Cancer: A Systematic Review and Meta-Analysis. Am. J. Roentgenol..

[15] Emmett L., Willowson K., Violet J., Shin J., Blanksby A., Lee J. (2017). Lutetium 177 PSMA Radionuclide Therapy for Men with Prostate Cancer: A Review of the Current Literature and Discussion of Practical Aspects of Therapy. J. Med. Radiat. Sci..

[16] Kratochwil C., Giesel F.L., Stefanova M., Benesova M., Bronzel M., Afshar-Oromieh A., Mier W., Eder M., Kopka K., Haberkorn U. (2016). PSMA-Targeted Radionuclide Therapy of Metastatic Castration-Resistant Prostate Cancer with 177Lu-Labeled PSMA-617. J. Nucl. Med..

[17] Hotta M., Gafita A., Czernin J., Calais J. (2022). Outcome of Patients with PSMA PET/CT Screen Failure by VISION Criteria and Treated with 177Lu-PSMA Therapy: A Multicenter Retrospective Analysis. J. Nucl. Med..

[18] Cook G.J.R., Wong W.-L., Sanghera B., Mangar S., Challapalli A., Bahl A., Bassett P., Leaning D., Schmidkonz C. (2022). Eligibility for 177 Lu-PSMA Therapy Depends on the Choice of Companion Diagnostic Tracer: A Comparison of 68 Ga-PSMA-11 and 99m Tc-MIP-1404 in Metastatic Castrate Resistant Prostate Cancer. J. Nucl. Med..

[19] Cao J., Chen Y., Hu M., Zhang W. (2021). Lu-PSMA-RLT of Metastatic Castration-Resistant Prostate Cancer: Limitations and Improvements. Ann. Nucl. Med..

[20] Hofman M.S., Violet J., Hicks R.J., Ferdinandus J., Ping Thang S., Akhurst T., Iravani A., Kong G., Ravi Kumar A., Murphy D.G. (2018). [^177^Lu]-PSMA-617 Radionuclide Treatment in Patients with Metastatic Castration-Resistant Prostate Cancer (LuPSMA Trial): A Single-Centre, Single-Arm, Phase 2 Study. Lancet Oncol..

[21] Vlachostergios P.J., Niaz M.J., Sun M., Mosallaie S.A., Thomas C., Christos P.J., Osborne J.R., Molina A.M., Nanus D.M., Bander N.H. (2021). Prostate-Specific Membrane Antigen Uptake and Survival in Metastatic Castration-Resistant Prostate Cancer. Front. Oncol..

[22] Thang S.P., Violet J., Sandhu S., Iravani A., Akhurst T., Kong G., Ravi Kumar A., Murphy D.G., Williams S.G., Hicks R.J. (2019). Poor Outcomes for Patients with Metastatic Castration-Resistant Prostate Cancer with Low Prostate-Specific Membrane Antigen (PSMA) Expression Deemed Ineligible for 177Lu-Labelled PSMA Radioligand Therapy. Eur. Urol. Oncol..

[23] Sjögreen Gleisner K., Chouin N., Gabina P.M., Cicone F., Gnesin S., Stokke C., Konijnenberg M., Cremonesi M., Verburg F.A., Bernhardt P. (2022). EANM Dosimetry Committee Recommendations for Dosimetry of 177Lu-Labelled Somatostatin-Receptor- and PSMA-Targeting Ligands. Eur. J. Nucl. Med. Mol. Imaging.

[24] Rosar F., Krause J., Bartholomä M., Maus S., Stemler T., Hierlmeier I., Linxweiler J., Ezziddin S., Khreish F. (2020). Efficacy and Safety of [225ac]Ac-Psma-617 Augmented [177lu]Lu-Psma-617 Radioligand Therapy in Patients with Highly Advanced Mcrpc with Poor Prognosis. Pharmaceutics.

[25] Khreish F., Ebert N., Ries M., Maus S., Rosar F., Bohnenberger H., Stemler T., Saar M., Bartholomä M., Ezziddin S. (2020). 225Ac-PSMA-617/177Lu-PSMA-617 Tandem Therapy of Metastatic Castration-Resistant Prostate Cancer: Pilot Experience. Eur. J. Nucl. Med. Mol. Imaging.

[26] Ruigrok E.A.M., Van Weerden W.M., Nonnekens J., De Jong M. (2019). The Future of PSMA-Targeted Radionuclide Therapy: An Overview of Recent Preclinical Research. Pharmaceutics.

[27] Benešová M., Bauder-Wüst U., Schäfer M., Klika K.D., Mier W., Haberkorn U., Kopka K., Eder M. (2016). Linker Modification Strategies to Control the Prostate-Specific Membrane Antigen (PSMA)-Targeting and Pharmacokinetic Properties of DOTA-Conjugated PSMA Inhibitors. J. Med. Chem..

[28] Mesters J.R., Barinka C., Li W., Tsukamoto T., Majer P., Slusher B.S., Konvalinka J., Hilgenfeld R. (2006). Structure of Glutamate Carboxypeptidase II, a Drug Target in Neuronal Damage and Prostate Cancer. EMBO J..

[29] Bařinka C., Rovenská M., Mlčochová P., Hlouchová K., Plechanovová A., Majer P., Tsukamoto T., Slusher B.S., Konvalinka J., Lubkowski J. (2007). Structural Insight into the Pharmacophore Pocket of Human Glutamate Carboxypeptidase II. J. Med. Chem..

[30] Kopka K., Benešová M., Bařinka C., Haberkorn U., Babich J. (2017). Glu-Ureido–Based Inhibitors of Prostate-Specific Membrane Antigen: Lessons Learned During the Development of a Novel Class of Low-Molecular-Weight Theranostic Radiotracers. J. Nucl. Med..

[31] Barrett J.A., Babich J.W., Zimmerman C.N., Maresca K.P., Hillier S.M., Eckelman W.C., Barone C., Femia F.J., Keith D., Joyal J.L. (2009). A Series of Halogenated Heterodimeric Inhibitors of Prostate Specific Membrane Antigen (PSMA) as Radiolabeled Probes for Targeting Prostate Cancer. J. Med. Chem..

[32] Hillier S.M., Maresca K.P., Femia F.J., Marquis J.C., Foss C.A., Nguyen N., Zimmerman C.N., Barrett J.A., Eckelman W.C., Pomper M.G. (2009). Preclinical Evaluation of Novel Glutamate-Urea-Lysine Analogues That Target Prostate-Specific Membrane Antigen as Molecular Imaging Pharmaceuticals for Prostate Cancer. Cancer Res..

[33] Kozikowski A.P., Nan F., Conti P., Zhang J., Ramadan E., Bzdega T., Wroblewska B., Neale J.H., Pshenichkin S., Wroblewski J.T. (2001). Design of Remarkably Simple, yet Potent Urea-Based Inhibitors of Glutamate Carboxypeptidase II (NAALADase). J. Med. Chem..

[34] Kularatne S.A., Zhou Z., Yang J., Post C.B., Low P.S. (2009). Design, Synthesis, and Preclinical Evaluation of Prostate-Specific Membrane Antigen Targeted 99mTc-Radioimaging Agents. Mol. Pharm..

[35] Benešová M., Umbricht C.A., Schibli R., Müller C. (2018). Albumin-Binding PSMA Ligands: Optimization of the Tissue Distribution Profile. Mol. Pharm..

[36] Wang G., Zang J., Jiang Y., Liu Q., Sui H., Wang R., Fan X., Zhang J., Zhu Z., Chen X. (2023). A Single-Arm, Low-Dose, Prospective Study of 177Lu-EB-PSMA Radioligand Therapy in Patients with Metastatic Castration-Resistant Prostate Cancer. J. Nucl. Med..

[37] Bendre S., Zhang Z., Kuo H.T., Rousseau J., Zhang C., Merkens H., Roxin Á., Bénard F., Lin K.S. (2020). Evaluation of Met-Val-Lys as a Renal Brush Border Enzyme-Cleavable Linker to Reduce Kidney Uptake of 68Ga-Labeled DOTA-Conjugated Peptides and Peptidomimetics. Molecules.

[38] Zia N.A., Cullinane C., Van Zuylekom J.K., Waldeck K., McInnes L.E., Buncic G., Haskali M.B., Roselt P.D., Hicks R.J., Donnelly P.S. (2019). A Bivalent Inhibitor of Prostate Specific Membrane Antigen Radiolabeled with Copper-64 with High Tumor Uptake and Retention. Angew. Chem. Int. Ed..

[39] Banerjee S.R., Pullambhatla M., Shallal H., Lisok A., Mease R.C., Pomper M.G. (2011). A Modular Strategy to Prepare Multivalent Inhibitors of Prostate-Specific Membrane Antigen (PSMA). Oncotarget.

[40] Schäfer M., Bauder-Wüst U., Leotta K., Zoller F., Mier W., Haberkorn U., Eisenhut M., Eder M. (2012). A Dimerized Urea-Based Inhibitor of the Prostatespecific Membrane Antigen For68Ga-PET Imaging of Prostate Cancer. EJNMMI Res..

[41] Wurzer A., Vágner A., Horváth D., Fellegi F., Wester H.J., Kálmán F.K., Notni J. (2018). Synthesis of Symmetrical Tetrameric Conjugates of the Radiolanthanide Chelator DOTPI for Application in Endoradiotherapy by Means of Click Chemistry. Front. Chem..

[42] Derks Y.H.W., Rijpkema M., Amatdjais-Groenen H.I.V., Kip A., Franssen G.M., Michiel Sedelaar J.P., Somford D.M., Simons M., Laverman P., Gotthardt M. (2021). Photosensitizer-Based Multimodal PSMA-Targeting Ligands for Intraoperative Detection of Prostate Cancer. Theranostics.

[43] Benesová M., Schäfer M., Bauder-Wüst U., Afshar-Oromieh A., Kratochwil C., Mier W., Haberkorn U., Kopka K., Eder M. (2015). Preclinical Evaluation of a Tailor-Made DOTA-Conjugated PSMA Inhibitor with Optimized Linker Moiety for Imaging and Endoradiotherapy of Prostate Cancer. J. Nucl. Med..

[44] Weineisen M., Simecek J., Schottelius M., Schwaiger M., Wester H.J. (2014). Synthesis and Preclinical Evaluation of DOTAGA-Conjugated PSMA Ligands for Functional Imaging and Endoradiotherapy of Prostate Cancer. EJNMMI Res..

[45] Brown S.P., Smith A.B. (2015). Peptide/Protein Stapling and Unstapling: Introduction of s-Tetrazine, Photochemical Release, and Regeneration of the Peptide/Protein. J. Am. Chem. Soc..

[46] De Galiza Barbosa F., Queiroz M.A., Nunes R.F., Costa L.B., Zaniboni E.C., Marin J.F.G., Cerri G.G., Buchpiguel C.A. (2020). Nonprostatic Diseases on PSMA PET Imaging: A Spectrum of Benign and Malignant Findings. Cancer Imaging.

[47] Zhang A.X., Murelli R.P., Barinka C., Michel J., Cocleaza A., Jorgensen W.L., Lubkowski J., Spiegel D.A. (2010). A Remote Arene-Binding Site on Prostate Specific Membrane Antigen Revealed by Antibody-Recruiting Small Molecules. J. Am. Chem. Soc..

[48] Chatalic K.L.S., Heskamp S., Konijnenberg M., Molkenboer-Kuenen J.D.M., Franssen G.M., Clahsen-van Groningen M.C., Schottelius M., Wester H.J., van Weerden W.M., Boerman O.C. (2016). Towards Personalized Treatment of Prostate Cancer: PSMA I&T, a Promising Prostate-Specific Membrane Antigen-Targeted Theranostic Agent. Theranostics.

[49] Wirtz M., Schmidt A., Schottelius M., Robu S., Günther T., Schwaiger M., Wester H.J. (2018). Synthesis and in Vitro and in Vivo Evaluation of Urea-Based PSMA Inhibitors with Increased Lipophilicity. EJNMMI Res..

[50] Huang S.S., Wang X., Zhang Y., Doke A., Difilippo F.P., Heston W.D. (2014). Improving the Biodistribution of PSMA-Targeting Tracers with a Highly Negatively Charged Linker. Prostate.

[51] Baranski A.C., Schäfer M., Bauder-Wüst U., Wacker A., Schmidt J., Liolios C., Mier W., Haberkorn U., Eisenhut M., Kopka K. (2017). Improving the Imaging Contrast of 68Ga-PSMA-11 by Targeted Linker Design: Charged Spacer Moieties Enhance the Pharmacokinetic Properties. Bioconjug. Chem..

[52] Suzuki C., Uehara T., Kanazawa N., Wada S., Suzuki H., Arano Y. (2018). Preferential Cleavage of a Tripeptide Linkage by Enzymes on Renal Brush Border Membrane to Reduce Renal Radioactivity Levels of Radiolabeled Antibody Fragments. J. Med. Chem..

[53] Selvaraj R., Fox J.M. (2013). Trans-Cyclooctene—A Stable, Voracious Dienophile for Bioorthogonal Labeling. Curr. Opin. Chem. Biol..

[54] Wüstemann T., Bauder-Wüst U., Schäfer M., Eder M., Benesova M., Leotta K., Kratochwil C., Haberkorn U., Kopka K., Mier W. (2016). Design of Internalizing PSMA-Specific Glu-Ureido-Based Radiotherapeuticals. Theranostics.

[55] Huang Y., Li H., Ye S., Tang G., Liang Y., Hu K. (2021). Synthesis and Preclinical Evaluation of an Al18F Radiofluorinated Bivalent PSMA Ligand. Eur. J. Med. Chem..

[56] Reissig F., Bauer D., Zarschler K., Novy Z., Bendova K., Ludik M.C., Kopka K., Pietzsch H.J., Petrik M., Mamat C. (2021). Towards Targeted Alpha Therapy with Actinium-225: Chelators for Mild Condition Radiolabeling and Targeting Psma—A Proof of Concept Study. Cancers.

[57] McInnes L.E., Cullinane C., Roselt P.D., Jackson S., Blyth B.J., van Dam E.M., Zia N.A., Harris M.J., Hicks R.J., Donnelly P.S. (2021). Therapeutic Efficacy of a Bivalent Inhibitor of Prostate-Specific Membrane Antigen Labeled with 67Cu. J. Nucl. Med..

[58] Kratochwil C., Giesel F.L., Leotta K., Eder M., Hoppe-Tich T., Youssoufian H., Kopka K., Babich J.W., Haberkorn U. (2015). PMPA for Nephroprotection in PSMA-Targeted Radionuclide Therapy of Prostate Cancer. J. Nucl. Med..

[59] Kalidindi T.M., Lee S.G., Jou K., Chakraborty G., Skafida M., Tagawa S.T., Bander N.H., Schoder H., Bodei L., Pandit-Taskar N. (2021). A Simple Strategy to Reduce the Salivary Gland and Kidney Uptake of PSMA-Targeting Small Molecule Radiopharmaceuticals. Eur. J. Nucl. Med. Mol. Imaging.

[60] Rousseau E., Lau J., Kuo H.T., Zhang Z., Merkens H., Hundal-Jabal N., Colpo N., Lin K.S., Bénard F. (2018). Monosodium Glutamate Reduces68Ga-PSMA-11 Uptake in Salivary Glands and Kidneys in a Preclinical Prostate Cancer Model. J. Nucl. Med..

[61] Srinivasan S., Yee N.A., Wu K., Zakharian M., Mahmoodi A., Royzen M., Oneto J.M.M. (2021). SQ3370 Activates Cytotoxic Drug via Click Chemistry at Tumor and Elicits Sustained Responses in Injected & Non-Injected Lesions. Adv. Ther..

[62] Van Duijnhoven S.M.J., Rossin R., Van Den Bosch S.M., Wheatcroft M.P., Hudson P.J., Robillard M.S. (2015). Diabody Pretargeting with Click Chemistry in Vivo. J. Nucl. Med..

[63] Handula M., Chen K.T., Seimbille Y. (2021). Iedda: An Attractive Bioorthogonal Reaction for Biomedical Applications. Molecules.

[64] Kuo H.-T., Lin K.-S., Zhang Z., Zhang C., Merkens H., Tan R., Roxin A., Uribe C.F., Bénard F. (2022). What a Difference a Methylene Makes: Replacing Glu with Asp or Aad in the Lys-Urea-Glu Pharmacophore of PSMA-Targeting Radioligands to Reduce Kidney and Salivary Gland Uptake. Theranostics.

[65] Felber V.B., Valentin M.A., Wester H.J. (2021). Design of PSMA Ligands with Modifications at the Inhibitor Part: An Approach to Reduce the Salivary Gland Uptake of Radiolabeled PSMA Inhibitors?. EJNMMI Radiopharm. Chem..

[66] Weller H.N., Rubin A.E., Moshiri B., Ruediger W., Li W.J., Allen J., Nolfo J., Bertok A., Rosso V.W. (2005). Development and Commercialization of the MiniBlock Synthesizer Family: A Historical Case Study. J. Lab. Autom..

[67] Eissler S., Kley M., Bächle D., Loidl G., Meier T., Samson D. (2017). Substitution Determination of Fmoc-Substituted Resins at Different Wavelengths. J. Pept. Sci..

[68] Breeman W.A.P., Chan H.S., de Blois E. (2014). Determination of Peptide Content and Purity of DOTA-Peptides by Metal Ion Titration and UPLC: An Alternative Method to Monitor Quality of DOTA-Peptides. J. Radioanal. Nucl. Chem..

[69] Chen K.-T., Nguyen K., Ieritano C., Gao F., Seimbille Y. (2018). A Flexible Synthesis of 68Ga-Labeled Carbonic Anhydrase IX (CAIX)-Targeted Molecules via CBT/1,2-Aminothiol Click Reaction. Molecules.

