# Supplementary material for: Preclinical Evaluation of a PSMA-Targeting Homodimer with an Optimized Linker for Imaging of Prostate Cancer
Source: Molecules. 2023 May 11;28(10):4022. doi: 10.3390/molecules28104022 (PMC10223019; doi:10.3390/molecules28104022)
Supplement: Supplementary file 1 [file molecules-28-04022-s001.zip › molecules-2385795-supplementary.pdf]

Supplementary Information

Preclinical evaluation of a PSMA-targeting homodimer with an optimized linker for imaging of prostate cancer

Erika Murce[1,2], Savanne Beekman[1,2], Evelien Spaan[1,2], Maryana Handula[1,2], Debra Stuurman[1,2], Corrina de Ridder[1,2] and Yann Seimbille[1,2,3,*].

[1]University Medical Center Rotterdam, Erasmus MC, Department of Radiology and Nuclear Medicine, Rotterdam, The Netherlands; e.murcesilva@erasmusmc.nl (E.M.); s.beekman@erasmusmc.nl (S.B.); e.spaan@erasmusmc.nl (E.S.); m.handula@erasmusmc.nl (M.H.); d.stuurman@erasmusmc.nl (D.S.); c.deridder@erasmusmc.nl (C.d.R.)

[2]Erasmus MC Cancer Institute, Rotterdam, The Netherlands

[3]TRIUMF, Life Sciences Division, Vancouver, Canada

[*]Corresponding author: y.seimbille@erasmusmc.nl

Table of Contents

Table of Contents 1
Chemical Synthesis of compounds **1** to **30** 1
Structure of compounds **1** to **23** 6
Characterization of compounds **1** to **30** 7
Summary of the library compounds **1** to **20** 17
Radio-HPLC chromatogram of [^{111}In]In-**22** 18
Radio-HPLC chromatogram of [^{111}In]In-**30** 18
Radio-HPLC chromatogram of [^{111}In]In-**27** 19
Stability of [^{111}In]In-**22** 19
Stability of [^{111}In]In-**30** 20
NAALADase Assay of PSMA-617, **22** and **30** 21
Uptake and internalization of [^{111}In]In-PSMA-617, [^{111}In]In-**22** and [^{111}In]In-**30** on LS174T cells 22
Ex vivo biodistribution data of [^{111}In]In-PSMA-617 22
Ex vivo biodistribution data of [^{111}In]In-**22** 23
Ex vivo biodistribution data of [^{111}In]In-**30** 24

Chemical Synthesis of compounds 1 to 30

EuK(Ahx) (1). Compound **1** was obtained by cleavage of the peptide bound to the tBu-O-Glu(tBu)-urea-Lys(Ahx)-resin, and purified by preparative HPLC (t_R = 1.41 min). **1** was obtained as a white solid (20.8 mg, 48 µmol, 69% yield), and was characterized by LC-MS (t_R = 1.99 min, purity = 94%). ESI-MS m/z: calc'd for $C_{18}H_{32}N_4O_8$ 432.22; found 433.20 $[M+H]^+$.

EuK(Ahx-Ala) (2). To 0.08 mmol of tBu-O-Glu(tBu)-urea-Lys(Ahx)-resin was added a solution of Fmoc-β-Ala-OH (99 mg, 0.32 mmol, 4.0 equiv.) in 2 mL DMF with 2-(1*H*-benzotriazol-1-yl)-1,1,3,3-tetramethyluronium hexafluorophosphate (HBTU, 118 mg, 0.31 mmol, 3.9 equiv.), Oxyma Pure (46 mg, 0.32 mmol, 4.0 equiv.) and DIPEA (139,2 µL, 0.80 mmol, 10.0 equiv.), and the resin was agitated for 45 minutes at room temperature

(rt). The Fmoc group was then removed using a solution of 4-methylpiperidine in DMF (10 mL; v/v, 1:4; 5+10 min, rt) and the compound was cleaved from the resin (2 mL TFA/TIS/H_2O (95:2.5:2.5) cocktail, 2 h, rt), then purified by preparative HPLC (t_R = 2.46 min). **2** was obtained as a white solid (3.3 mg, 6.5 µmol, 8% yield), and was characterized by LC-MS (t_R = 3.10 min, purity > 90%). ESI-MS m/z: calc'd for $C_{21}H_{37}N_5O_9$ 503.26; found 504.30 $[M+H]^+$.

EuK (Ahx-Ala-Ala) (3). To 0.08 mmol of tBu-O-Glu(tBu)-urea-Lys(Ahx)-resin was added a solution of Fmoc-β-Ala-OH (99 mg, 0.32 mmol, 4.0 equiv.) in 2 mL DMF with HBTU (118 mg, 0.31 mmol, 3.9 equiv.), Oxyma Pure (46 mg, 0.32 mmol, 4.0 equiv.) and DIPEA (139,2 µL, 0.80 mmol, 10.0 equiv.), and the resin was agitated for 45 minutes at rt. The Fmoc group was then removed and another coupling with Fmoc-β-Ala-OH was performed, as described above. After Fmoc deprotection, the compound was cleaved from the resin and purified by preparative HPLC (t_R = 2.45 min). **3** was obtained as a white solid (18.5 mg, 32.2 µmol, 40% yield), and was characterized by LC-MS (t_R = 2.92 min, purity > 95%). ESI-MS m/z: calc'd for $C_{24}H_{42}N_6O_{10}$ 574.30; found 575.30 $[M+H]^+$.

EuK(Ahx-Ala-Phe) (4). 4 was prepared according to the method described above for **3**, by conjugating Fmoc-β-Ala-OH and Fmoc-Phe-OH. After cleavage from the resin and purification by preparative HPLC (t_R = 2.74 min), **4** was obtained as a white solid (21.6 mg, 33.2 µmol, 42% yield), and was characterized by LC-MS (t_R = 3.26 min, purity > 95%). ESI-MS m/z: calc'd for $C_{30}H_{46}N_6O_{10}$ 650.33; found 651.30 $[M+H]^+$.

EuK(Ahx-Ala-Sta) (5). 5 was prepared according to the method described above for **3**, by conjugating Fmoc-β-Ala-OH and Fmoc-Sta-OH. After cleavage from the resin and purification by preparative HPLC (t_R = 2.56 min), **5** was obtained as a white solid (15.9 mg, 24 µmol, 30% yield), and was characterized by LC-MS (t_R = 3.15 min, purity > 95%). ESI-MS m/z: calc'd for $C_{29}H_{52}N_6O_{11}$ 660.37; found 661.40 $[M+H]^+$.

EuK(Ahx-Phe) (6). 6 was prepared according to the method described above for **3**, by conjugating Fmoc-Phe-OH. After cleavage from the resin and purification by preparative HPLC (t_R = 2.81 min) as a white solid (22.7 mg, 39 µmol, 49% yield), and was characterized by LC-MS (t_R = 3.36 min, purity > 95%). ESI-MS m/z: calc'd for $C_{27}H_{41}N_5O_9$ 579.29; found 580.30. $[M+H]^+$.

EuK(Ahx-Phe-Phe) (7). **7** was prepared according to the method described above for **3**, by conjugating Fmoc-Phe-OH two times successively. After cleavage from the resin and purification by preparative HPLC (t_R = 3.22 min), **7** was obtained as a white solid (27.0 mg, 37 µmol, 47% yield), and was characterized by LC-MS (t_R = 3.65 min, purity > 90%). ESI-MS m/z: calc'd for $C_{36}H_{50}N_6O_{10}$ 726.36; found 727.30 $[M+H]^+$.

EuK(Ahx-Phe-Ala) (8). **8** was prepared according to the method described above for **3**, by conjugating Fmoc-Phe-OH and Fmoc-β-Ala-OH. After cleavage from the resin and purification by preparative HPLC (t_R = 2.91 min), **8** was obtained as a white solid (21.0 mg, 32.2 μmol, 40% yield), and was characterized by LC-MS (t_R = 3.40 min, purity > 90%). ESI-MS m/z: calc'd for $C_{30}H_{46}N_6O_{10}$ 650.33; found 651.30 $[M+H]^+$.

EuK(Ahx-Phe-Sta) (9). **9** was prepared according to the method described above for **3**, by conjugating Fmoc-Phe-OH and Fmoc-Sta-OH. After cleavage from the resin and purification by preparative HPLC (t_R = 3.22 min), **9** was obtained as a white solid (17.0 mg, 23 μmol, 29% yield), and was characterized by LC-MS (t_R = 3.63 min, purity > 90%). ESI-MS m/z: calc'd for $C_{35}H_{56}N_6O_{11}$ 736.40; found 737.40 $[M+H]^+$.

EuK(Ahx-Sta) (10). **10** was prepared according to the method described above for **3**, by conjugating Fmoc-Sta-OH. After cleavage from the resin and purification by preparative HPLC (t_R = 2.92 min), **10** was obtained as a white solid (9.9 mg, 16.7 μmol, 21% yield), and was characterized by LC-MS (t_R = 3.20 min, purity = 92%). ESI-MS m/z: calc'd for $C_{26}H_{47}N_5O_{10}$ 589.33; found 590.30 $[M+H]^+$.

EuK(Ahx-Sta-Sta) (11). **11** was prepared according to the method described above for **3**, by conjugating Fmoc-Sta-OH two times successively. After cleavage from the resin and purification by preparative HPLC (t_R = 2.88 min), **11** was obtained as a white solid (3.6 mg, 4.8 μmol, 6% yield), and was characterized by LC-MS (t_R = 3.53 min, purity > 95%). ESI-MS m/z: calc'd for $C_{34}H_{62}N_6O_{12}$ 746.44; found 747.40 $[M+H]^+$.

EuK(Ahx-Sta-Phe) (12). **12** was prepared according to the method described above for **3**, by conjugating Fmoc-Sta-OH and Fmoc-Phe-OH. After cleavage from the resin and purification by preparative HPLC (t_R = 2.94 min), **12** was obtained as a white solid (4.4 mg, 5.9 μmol, 7.5% yield) and was characterized by LC-MS (t_R = 3.49 min, purity = 94%). ESI-MS m/z: calc'd for $C_{35}H_{56}N_6O_{11}$ 736.40; found 737.30 $[M+H]^+$.

EuK(Ahx-Sta-Ala) (13). **13** was prepared according to the method described above for **3**, by conjugating Fmoc-Sta-OH and Fmoc-β-Ala-OH. After cleavage from the resin and purification by preparative HPLC (t_R = 2.65 min), **13** was obtained as a white solid (2.3 mg, 3.5 μmol, 4.5% yield), and was characterized by LC-MS (t_R = 3.36 min, purity = 90%). ESI-MS m/z: calc'd for $C_{29}H_{52}N_6O_{11}$ 660.37; found 661.30 $[M+H]^+$.

EuK(Ahx-Sta-Phe-DOTA) (14). To 0.05 mmol of compound **12** in solid phase was added a solution of DOTA-tris(*t*Bu) ester (0.15 mmol, 86 mg, 3.0 equiv.), PyBOP (0.15 mmol, 78 mg, 3.0 equiv.) and DIPEA (0.3 mmol, 53 μL, 6.0 equiv.) in 2 mL DMF. The reaction was monitored by LC-MS. Compound **14** was then obtained after cleavage from the resin and purification by preparative HPLC (t_R = 3.16 min) as a white solid (4.5 mg, 4 μmol,

8% yield), and was characterized by LC-MS (t_R = 3.52 min, purity = 94%). ESI-MS m/z: calc'd for $C_{51}H_{82}N_{10}O_{18}$ 1122.58; found 1123.50 $[M+H]^+$.

EuK(Ahx-Sta-Phe-α-Dap) (15). To 0.05 mmol of compound **12** in solid phase was added a solution of Fmoc-Dap(Boc)-OH (85.2 mg, 0.20 mmol, 4 equiv.) in 2 mL DMF with HBTU (74 mg, 0.19 mmol, 3.9 equiv.), Oxyma Pure (28.4 mg, 0.20 mmol, 4 equiv.) and DIPEA (87 µL, 0.50 mmol, 10 equiv.), and the resin was agitated for 45 minutes at rt. The Fmoc group was then removed and compound **15** was obtained after cleavage from the resin and purification by preparative HPLC (t_R = 2.82 min) as a white solid (16.3 mg, 19.8 µmol, 39% yield), and was characterized by LC-MS (t_R = 3.14 min, purity > 95%). ESI-MS m/z: calc'd for $C_{38}H_{62}N_8O_{12}$ 822.45; found 823.40 $[M+H]^+$.

EuK(Ahx-Sta-Phe-Asp) (16). 16 was prepared according to the method described above for **15**, by conjugating Fmoc-Asp(tBu)-OH. After cleavage from the resin and purification by preparative HPLC (t_R = 3.30 min), **16** was obtained as a white solid (11.1 mg, 13 µmol, 26% yield), and was characterized by LC-MS (t_R = 3.64 min, purity > 95%). ESI-MS m/z: calc'd for $C_{39}H_{61}N_7O_{14}$ 851.43; found 852.40 $[M+H]^+$.

EuK(Ahx-Sta-Phe-Tyr) (17). 17 was prepared according to the method described above for **15**, by conjugating Fmoc-Tyr(tBu)-OH. After cleavage from the resin and purification by preparative HPLC (t_R = 3.26 min), **17** was obtained as a white solid (17.6 mg, 19 µmol, 38% yield), and was characterized by LC-MS (t_R = 3.63 min, purity > 90%). ESI-MS m/z: calc'd for $C_{44}H_{65}N_7O_{13}$ 899.46; found 900.40 $[M+H]^+$.

EuK(Ahx-Sta-Phe-Phe) (18). 18 was prepared according to the method described above for **15**, by conjugating Fmoc-Phe-OH. After cleavage from the resin and purification by preparative HPLC (t_R = 3.46 min), **18** was obtained as a white solid (9.0 mg, 10 µmol, 20% yield), and was characterized by LC-MS (t_R = 3.87 min, purity > 95%). ESI-MS m/z: calc'd for $C_{44}H_{65}N_7O_{12}$ 883.47; found 884.40 $[M+H]^+$.

EuK(Ahx-Sta-Phe-Sta) (19). 19 was prepared according to the method described above for **15**, by conjugating Fmoc-Sta-OH. After cleavage from the resin and purification by preparative HPLC (t_R = 3.42 min), **19** was obtained as a white solid (10.6 mg, 11.8 µmol, 23.6% yield), and was characterized by LC-MS (t_R = 3.79 min, purity = 95%). ESI-MS m/z: calc'd for $C_{43}H_{71}N_7O_{13}$ 893.51; found 894.50 $[M+H]^+$.

EuK(Ahx-Sta-Phe-β-Dap) (20). 20 was prepared according to the method described above for **15**, by conjugating Boc-Dap(Fmoc)-OH. After cleavage from the resin and purification by preparative HPLC (t_R = 2.72

min), **20** was obtained as a white solid (11.3 mg, 13.7 μmol, 27% yield), and was characterized by LC-MS (t_R = 3.26 min, purity > 95%). ESI-MS m/z: calc'd for $C_{38}H_{62}N_8O_{12}$ 822.45; found 823.40 $[M+H]^+$.

EuK(Ahx-Sta-D-Phe-Asp) (21). 21 was prepared according to the method described above for **3**, by conjugating Fmoc-Sta-OH, Fmoc-D-Phe-OH and Fmoc-Asp(tBu)-OH**.** Compound **21** was obtained after final Fmoc deprotection as a resin-bound intermediate, and was further used without purification.

EuK(Ahx-Sta-D-Phe-Asp-DOTA-GA) (23). 23 was prepared according to the method described above for **22**, starting from compound **21.** Purification by preparative HPLC (t_R = 3.62 min) yielded **23** as a pure white solid (24.2 mg, 18.4 μmol, 37% yield), which was characterized by LC-MS (t_R = 3.79 min, purity > 95%). ESI-MS m/z: calc'd for $C_{58}H_{91}N_{11}O_{23}$ 1309.63; found 1310.60 $[M+H]^+$.

EuK(Ahx-Sta-Phe-Asp-(CH$_2$)$_3$-SH) (24). To compound **16** in solid phase was added a solution of 3-(tritylthio)propionic acid (70 mg, 0.20 mmol, 4.0 equiv.) in 2 mL DMF with HBTU (74 mg, 0.19 mmol, 3.9 equiv.), Oxyma Pure (28.4 mg, 0.20 mmol, 4.0 equiv.) and DIPEA (87 μL, 0.50 mmol, 10.0 equiv.), and the resin was agitated for 45 minutes at rt. After cleavage from the resin and purification by preparative HPLC (t_R = 4.36 min), **24** was obtained as a white solid (15.7 mg, 16.7 μmol, 33% yield), and was characterized by LC-MS (t_R = 4.24 min, purity = 95%). ESI-MS m/z: calc'd for $C_{42}H_{65}N_7O_{15}S$ 939.43; found 940.30 $[M+H]^+$.

[EuK(Ahx-Sta-L-Phe-Asp-(CH$_2$)$_3$-SH)]$_2$-Tz (25). 7.3 mg of **24** (7.7 μmol, 1.0 equiv.) was solubilised in 3.75 mL of a solution of 50 mM NaH_2PO_4 (pH 5). A solution of 3,6-Dichloro-1,2,4,5-tetrazine (3.4 mg, 23.1 μmol, 3.0 equiv.) in $CHCl_3$ (3.75 mL) was added to **24** and both phases were stirred vigorously for 1 minute. The mixture was centrifuged for 1 minute at 2500 RPM and the aqueous phase was collected. The organic layer was extracted with an additional portion of water, then centrifuged once more at 2500 RPM for 1 minute. The combined water fractions were lyophilized and the crude was then purified by HPLC (t_R = 4.77 min). **25** was obtained as a pink solid (2.2 mg, 1.2 μmol, 30% yield), and was characterized by LC-MS (t_R = 4.56 min, purity > 95%). ESI-MS m/z: calc'd for $C_{86}H_{128}N_{18}O_{30}S_2$ 1958.19; found 1958.90 $[M+H]^+$.

[EuK(Ahx-Sta-L-Phe-Asp-(CH$_2$)$_3$-SH)]$_2$-Tz-TCO-DOTA (27). 25 (2.2 mg, 1.1 μmol, 1.0 equiv.) was solubilized in H_2O/ACN (1:1)**.** A solution of **26** (67 μL, 10 mg/mL, 1.0 equiv.) in H_2O/ACN (1:1) was added to the solution of **25** and the reaction was stirred at 37°C for 30 minutes. The orange-pink solution became colorless when the reaction was complete. The solution was lyophilized and purified by HPLC (t_R = 4.17 min) to yield **27,** as a white solid (0.8 mg, 0.31 umol, 34% yield), which was characterized by LC-MS (t_R = 4.23 min, purity > 95%). ESI-MS m/z: calc'd for $C_{114}H_{176}N_{22}O_{39}S_2$ 2542.90.; found 2543.60 $[M+H]^+$; 1271.90 $[M+2H]^{2+}$.

Structure of compounds **1** to **23**

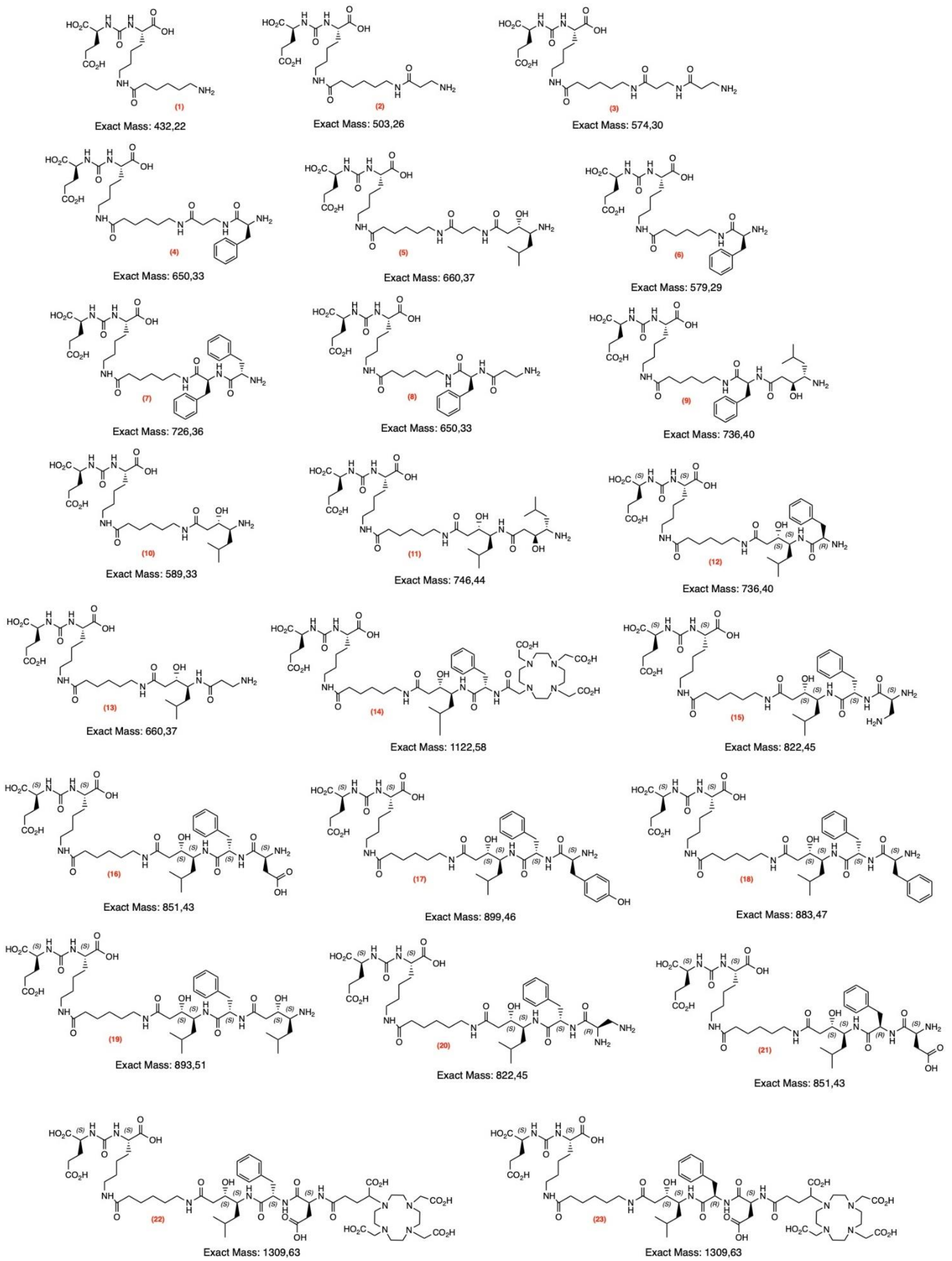

Figure S1. Structure of compounds **1** to **23**.

Characterization of compounds **1** to **30**

The library compounds **1-20** were obtained after HPLC purification in chemical purity >90% as verified by LC-MS. They were visualized at 220 nm and identified by the ESI-MS.

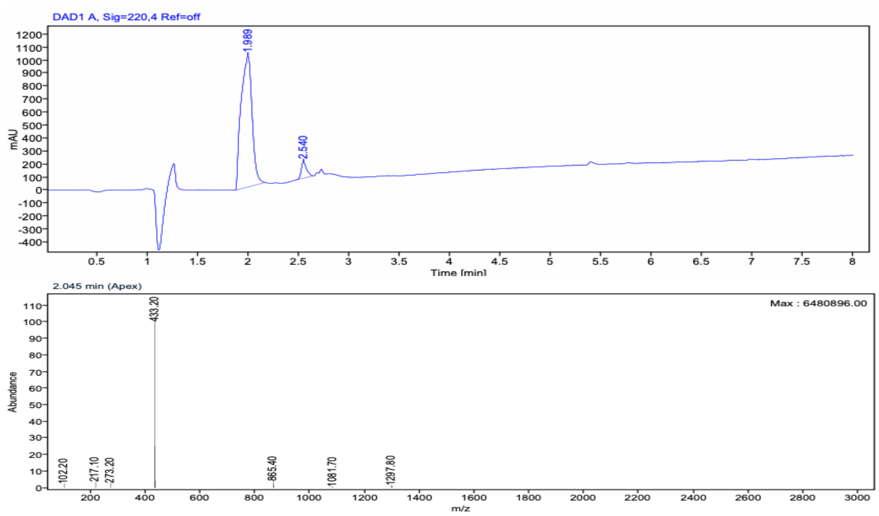

Figure S2.1: LC chromatogram and mass spectra of **1**.

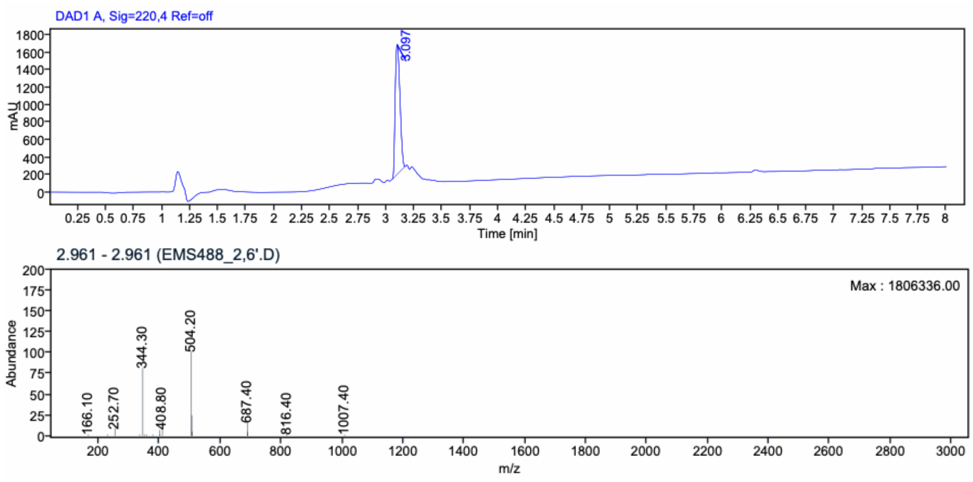

Figure S2.2: LC chromatogram and mass spectra of **2**.

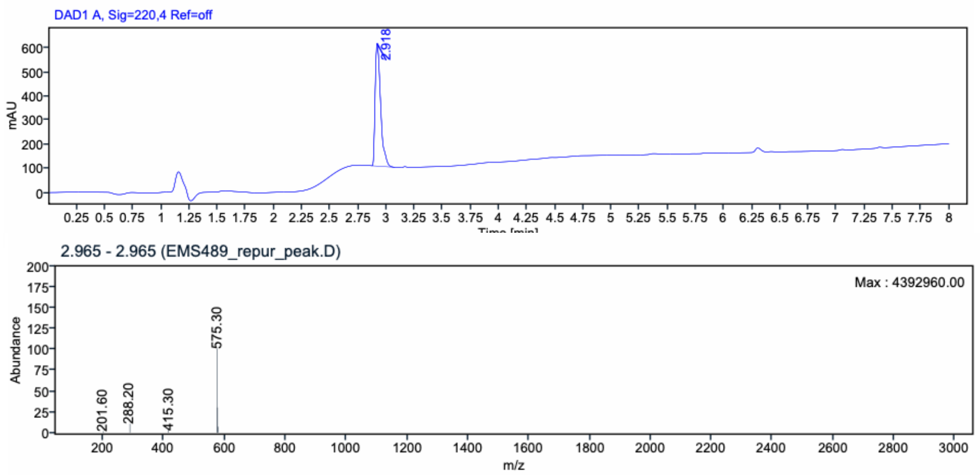

Figure S2.3: LC chromatogram and mass spectra of **3**.

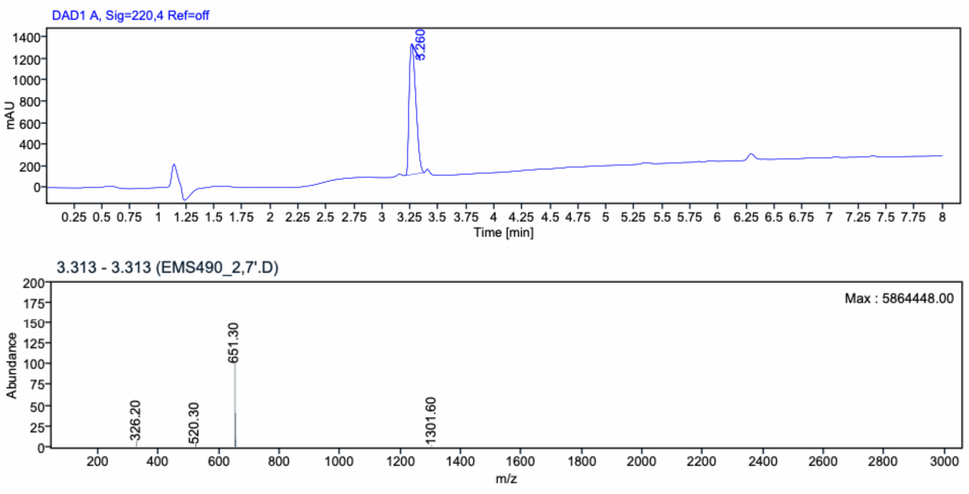

Figure S2.4: LC chromatogram and mass spectra of **4**.

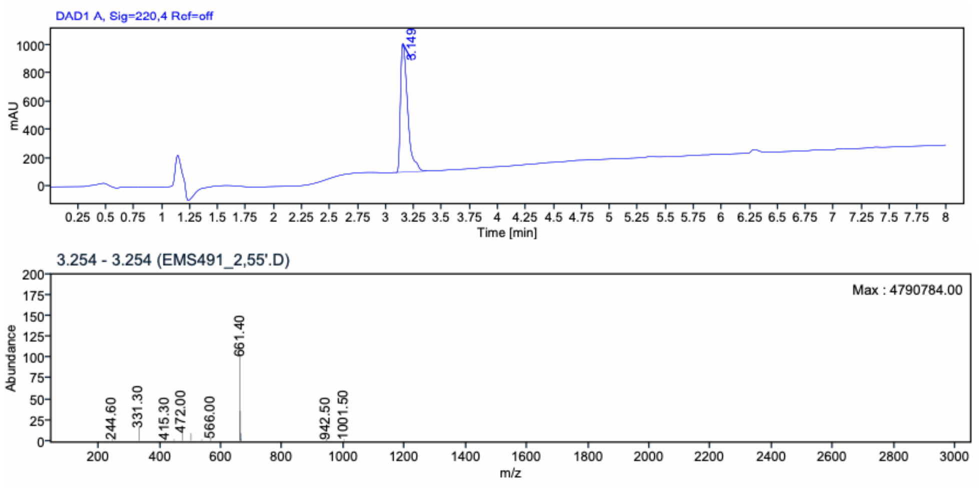

Figure S2.5: LC chromatogram and mass spectra of **5**.

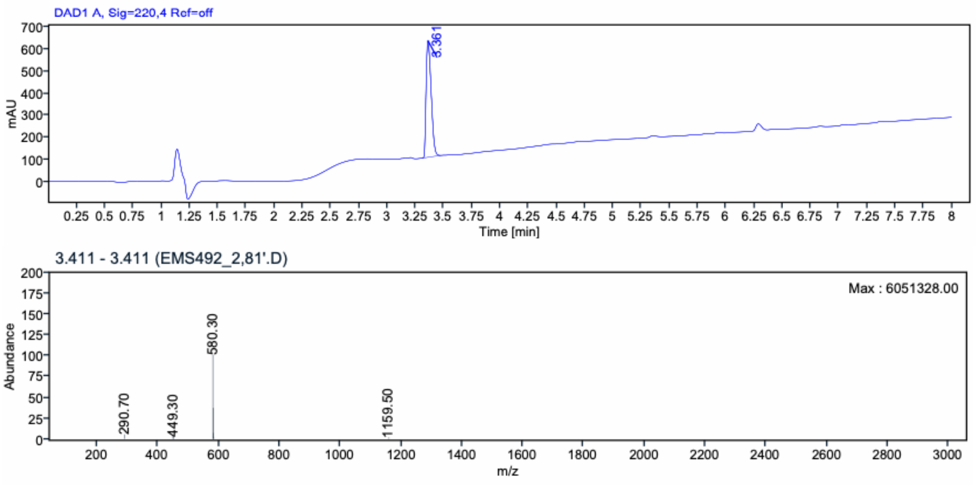

Figure S2.6: LC chromatogram and mass spectra of **6**.

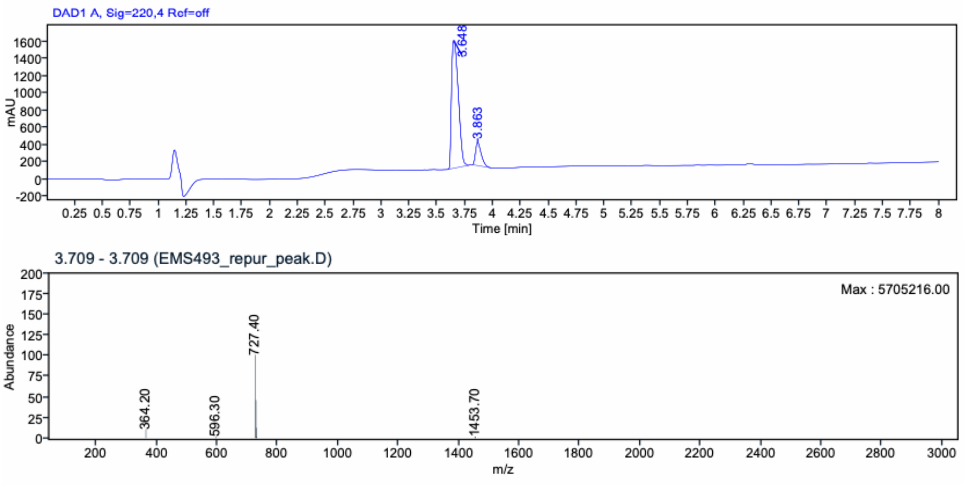

Figure S2.7: LC chromatogram and mass spectra of **7**.

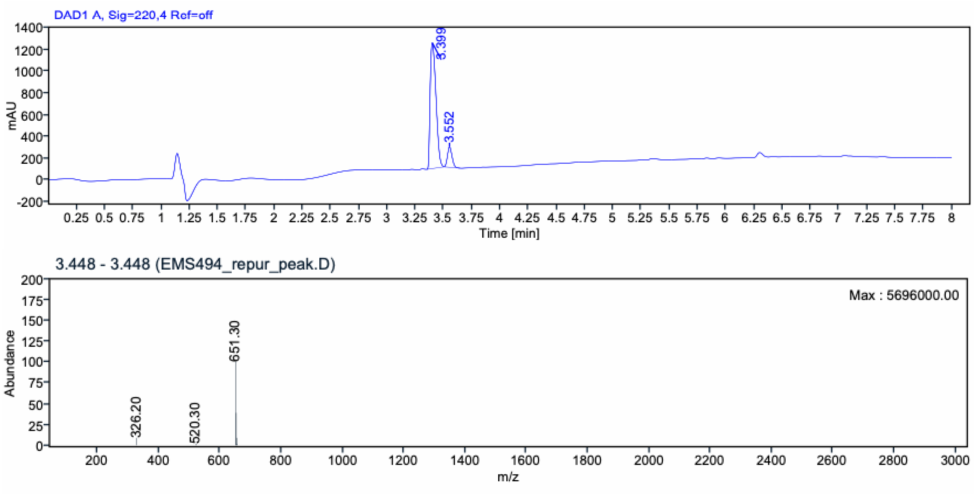

Figure S2.8: LC chromatogram and mass spectra of **8**.

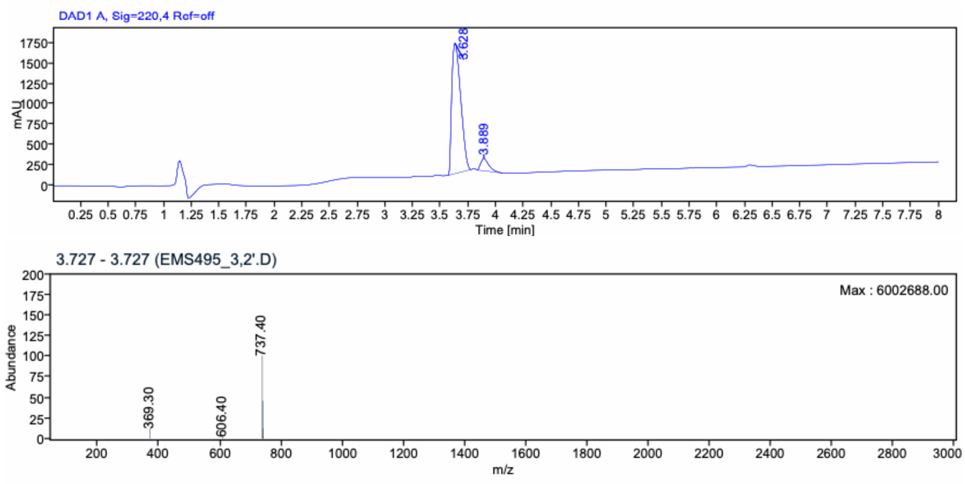

Figure S2.9: LC chromatogram and mass spectra of **9**.

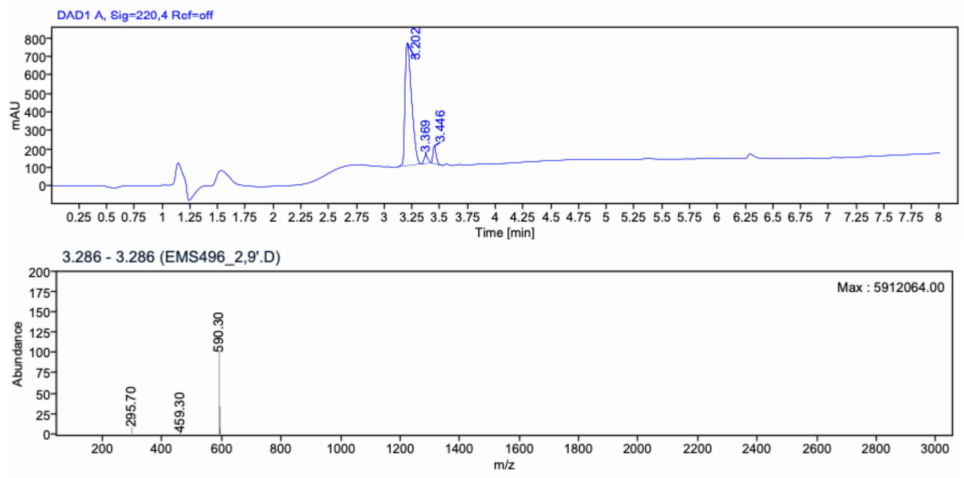

Figure S2.10: LC chromatogram and mass spectra of **10**.

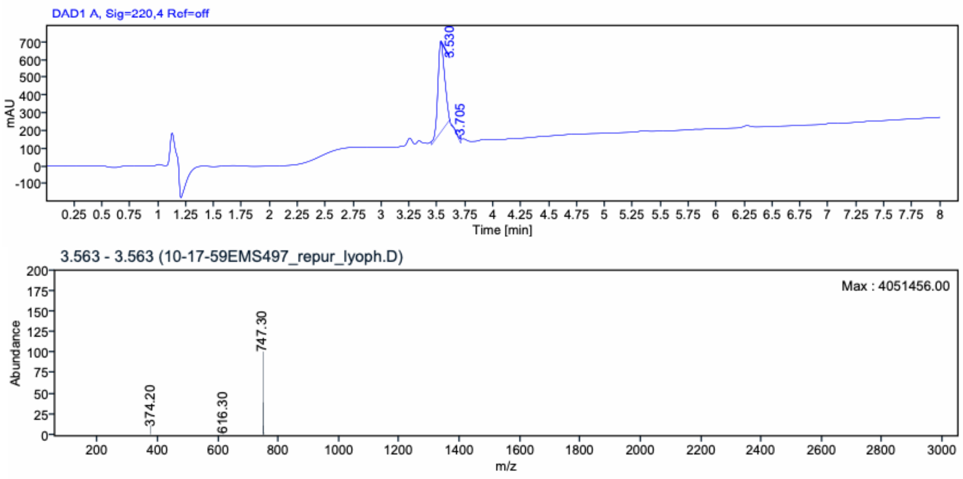

Figure S2.11: LC chromatogram and mass spectra of **11**.

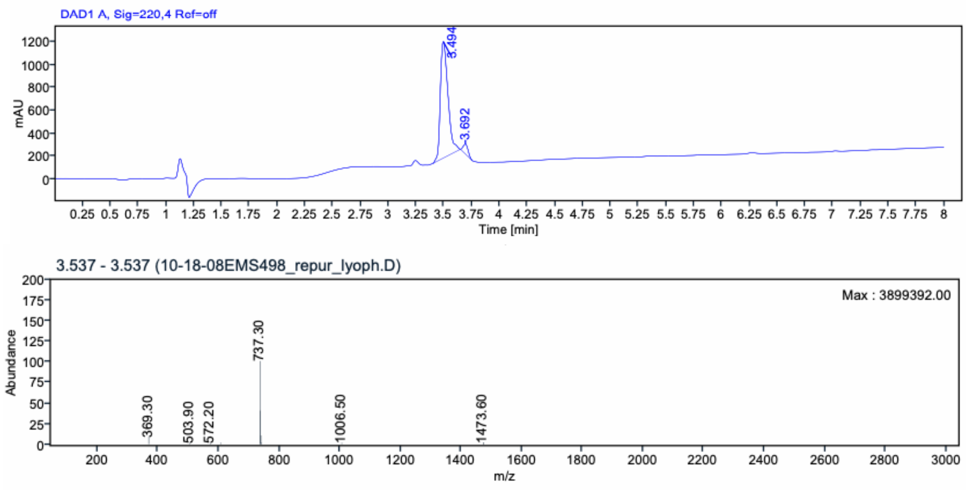

Figure S2.12: LC chromatogram and mass spectra of **12**.

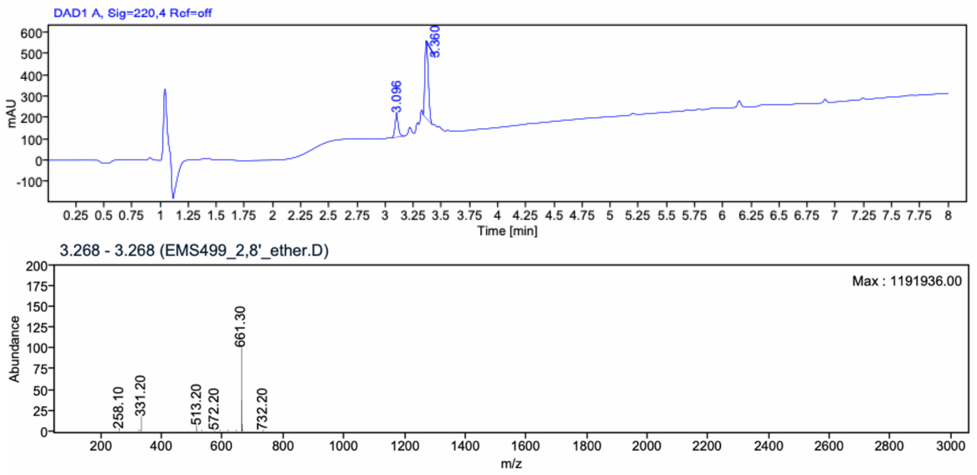

Figure S2.13: LC chromatogram and mass spectra of **13**.

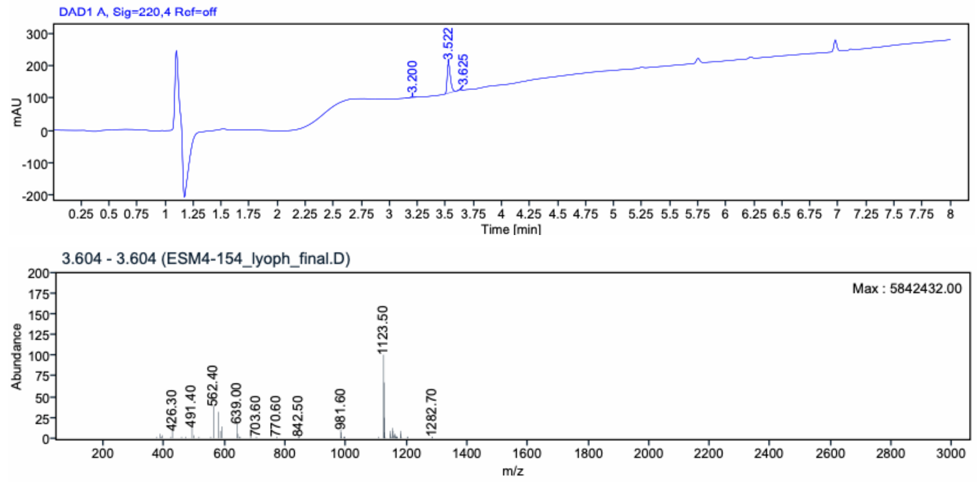

Figure S2.14: LC chromatogram and mass spectra of **14**.

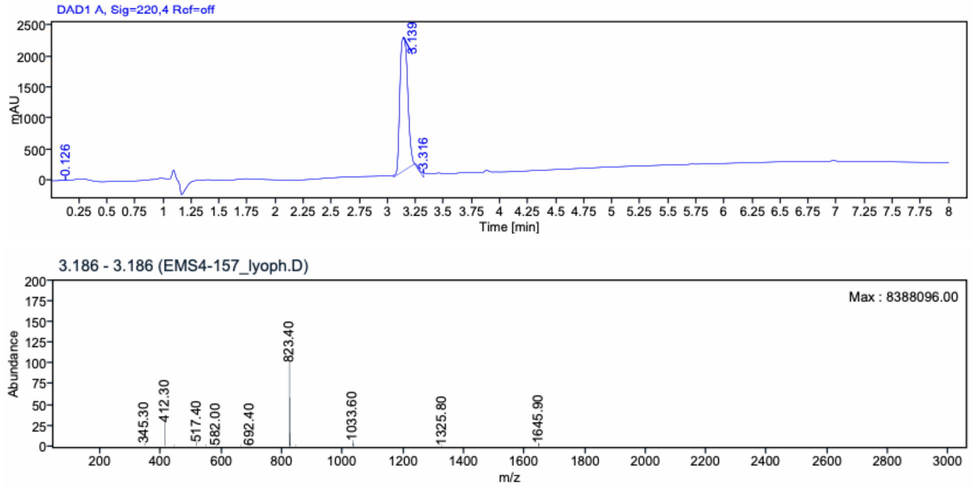

Figure S2.15: LC chromatogram and mass spectra of **15**.

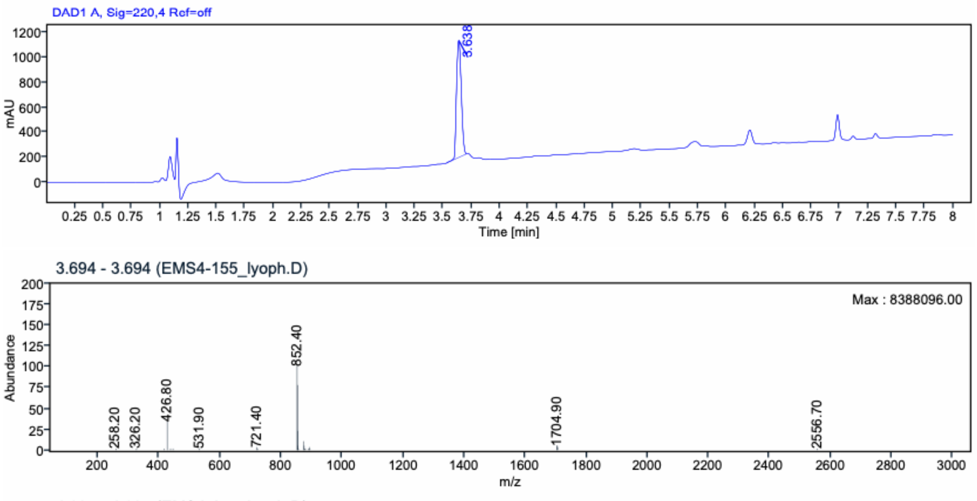

Figure S2.16: LC chromatogram and mass spectra of **16**.

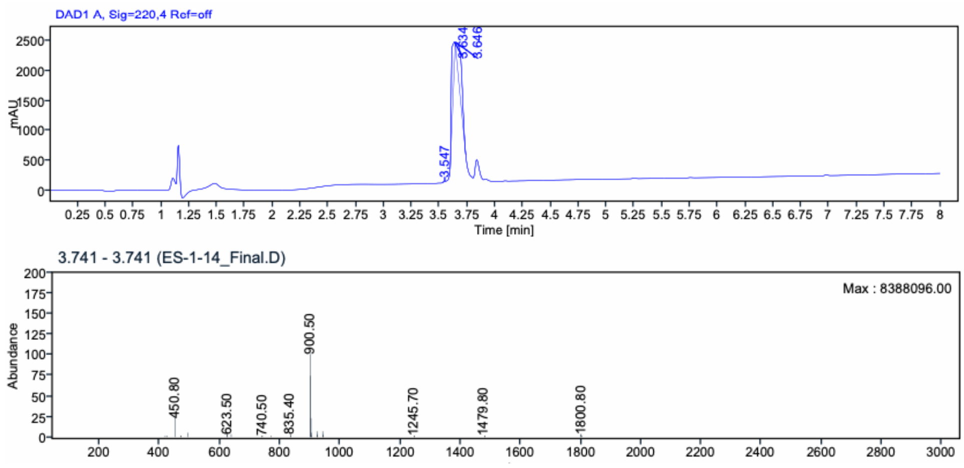

Figure S2.17: LC chromatogram and mass spectra of **17**.

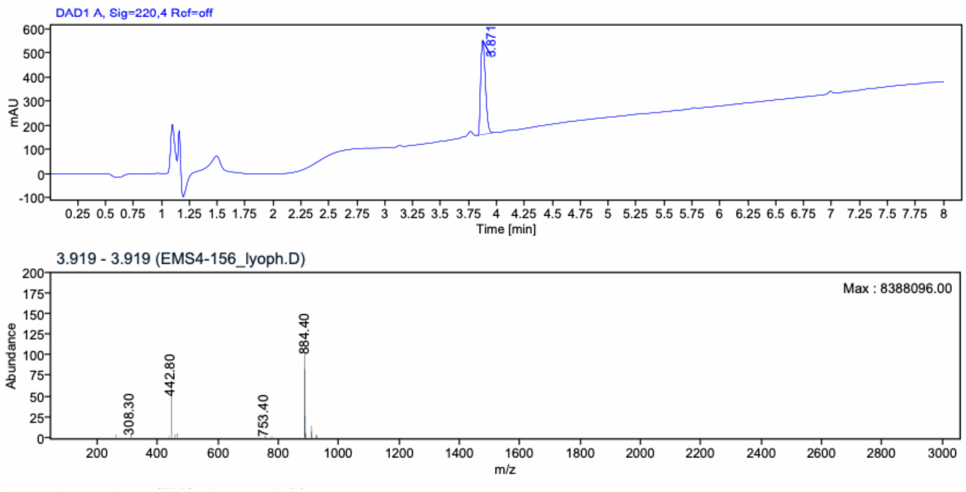

Figure S2.18: LC chromatogram and mass spectra of **18**.

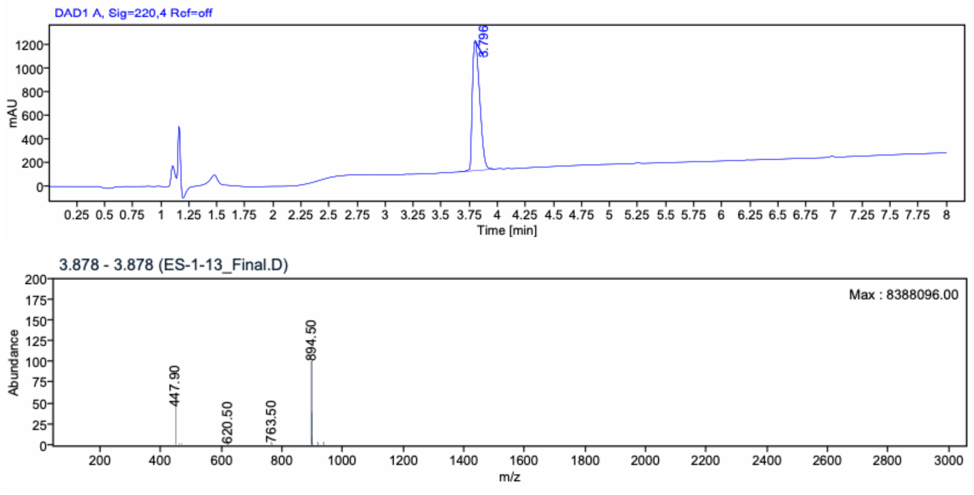

Figure S2.19: LC chromatogram and mass spectra of **19.**

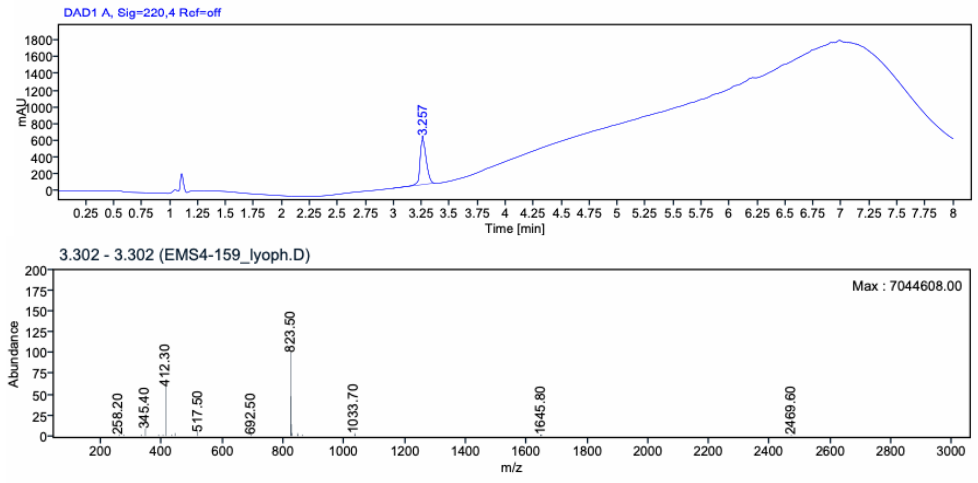

Figure S2.20: LC chromatogram and mass spectra of **20.**

Compounds **22**-**30** were obtained after HPLC purification in chemical purity >95% as verified by LC-MS.

They were identified by the ESI-MS and their UV signals were monitored at either 220 nm or 254 nm.

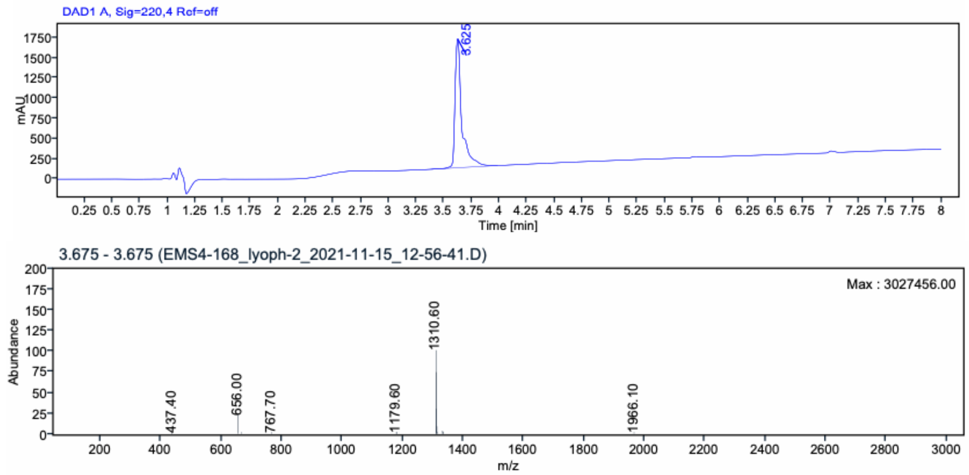

Figure S2.22: LC chromatogram and mass spectra of **22.**

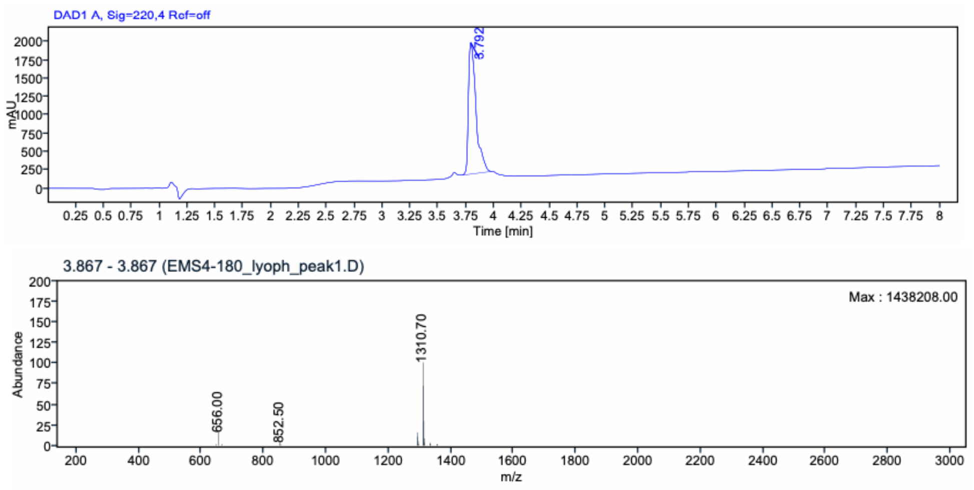

Figure S2.23: LC chromatogram and mass spectra of **23.**

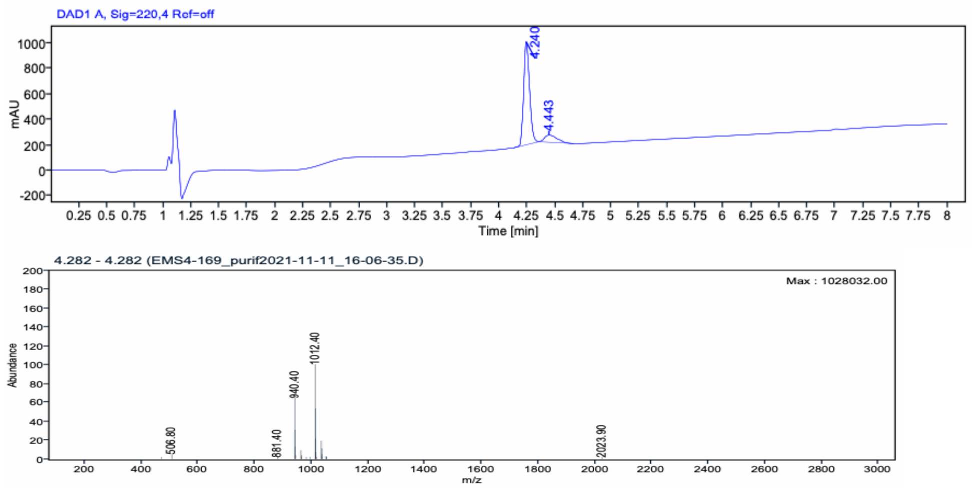

Figure S2.24: LC chromatogram and mass spectra of **24.**

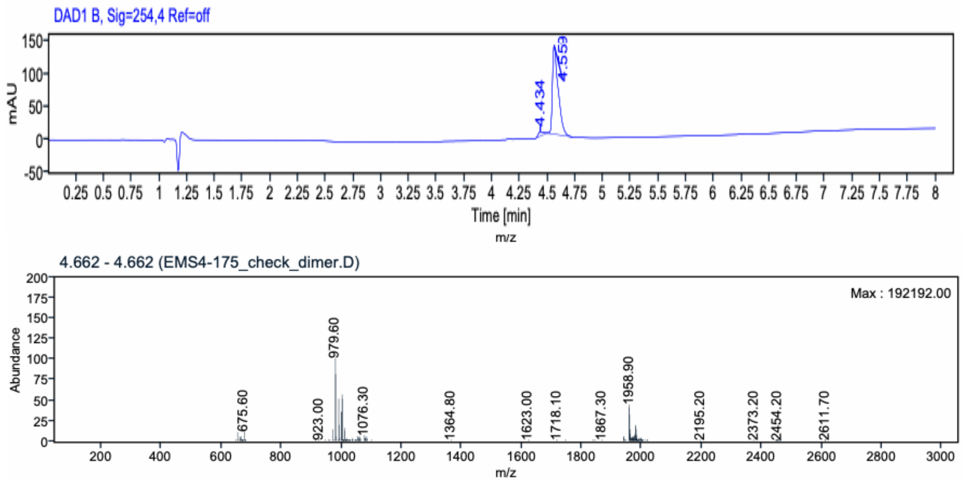

Figure S3.25: LC chromatogram and mass spectra of **25.**

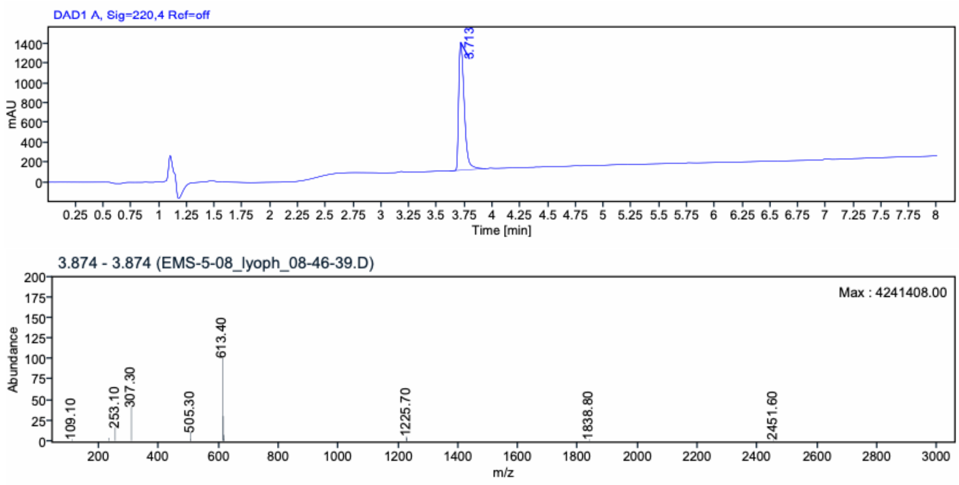

Figure S2.26: LC chromatogram and mass spectra of **26.**

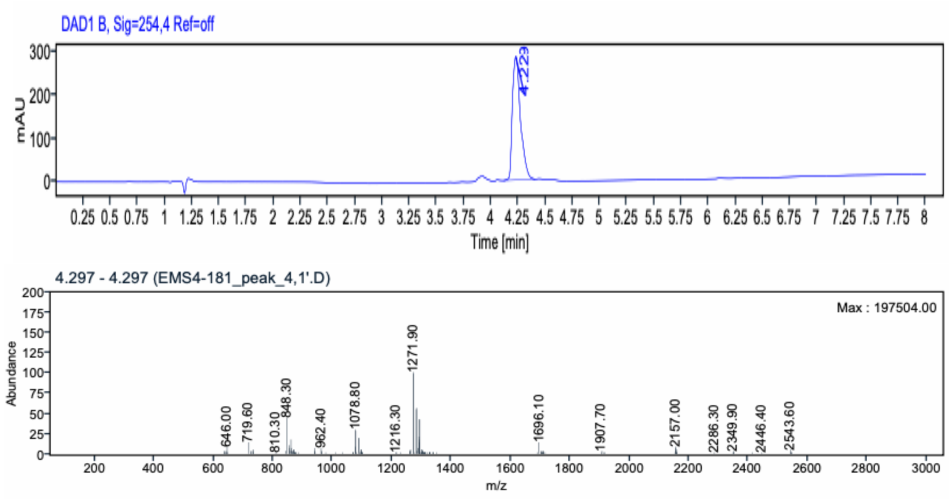

Figure S2.27: LC chromatogram and mass spectra of **27.**

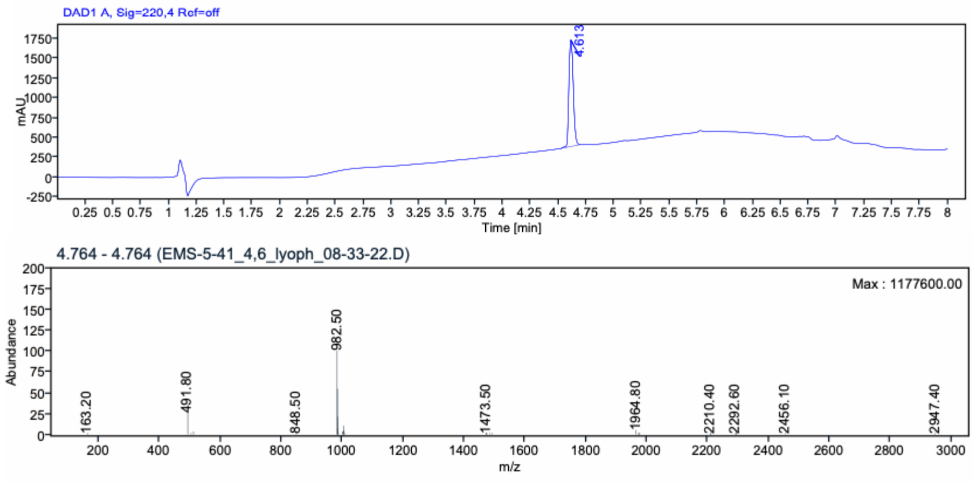

Figure S2.28: LC chromatogram and mass spectra of **28.**

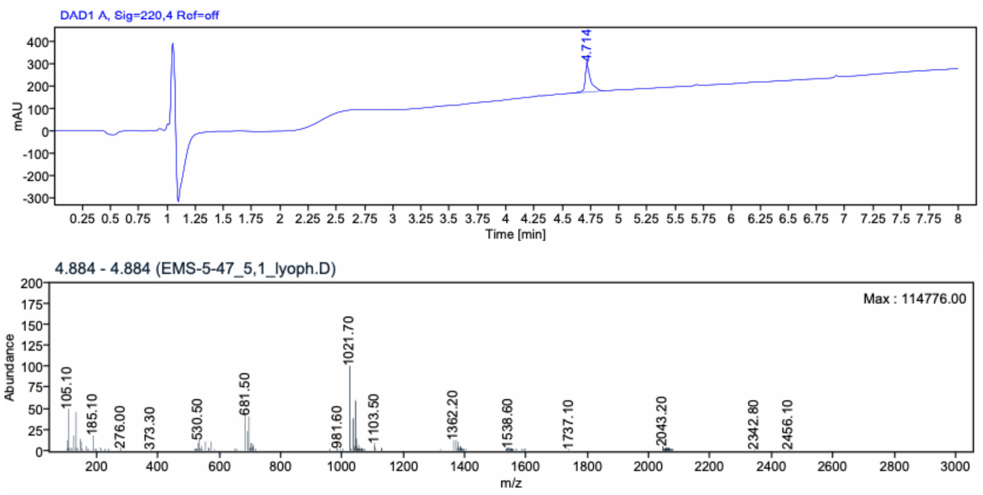

Figure S2.29: LC chromatogram and mass spectra of **29.**

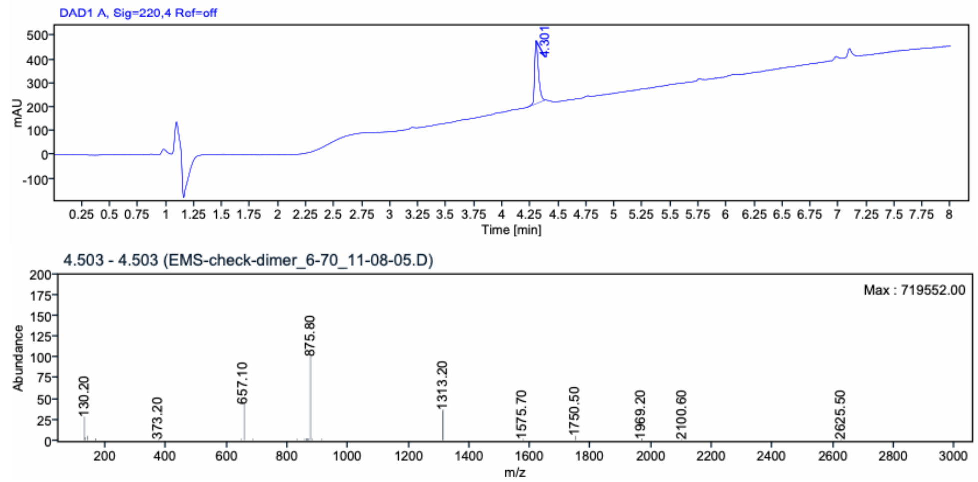

Figure S2.30: LC chromatogram and mass spectra of **30.**

Summary of the library compounds **1** to **20**

Table S1. Summary of the properties of compounds **1-20**, including the chemical yield after synthesis and HPLC purification, molecular weight, value obtained from the mass spectrometry, HPLC (for purification) and LC (for analysis of the purity) retention time and IC_{50} obtained via the NAALADase enzymatic assay.

Compound	Yield (%)	Exact Mass	ESI-MS ($[M+H]^+$)	Retention time (LC, min)	Retention time (HPLC, min)	IC_{50}, nM (NAALADase)
**1** (Ahx)	69%	432.22	433.10	1.989	1.408	9.86 ± 1.7
**2** (Ahx-Ala)	8%	503.26	504.30	3.097	2.458	23.1 ± 3.1
**3** (Ahx-Ala-Ala)	40%	574.30	575.30	2.783	2.455	20.1 ± 2.7
**4** (Ahx-Ala-Phe)	42%	650.33	651.30	3.248	2.740	2.86 ± 0.3
**5** (Ahx-Ala-Sta)	30%	660.37	661.40	3.131	2.559	10.1 ± 3.2
**6** (Ahx-Phe)	49%	579.29	580.30	3.281	2.809	3.17 ± 0.5
**7** (Ahx-Phe-Phe)	47%	726.36	727.30	3.657	3.218	1.72 ± 0.4
**8** (Ahx-Phe-Ala)	40%	650.33	651.30	3.384	2.915	4.53 ± 0.4
**9** (Ahx-Phe-Sta)	29%	736.40	737.40	3.645	3.225	7.00 ± 0.3
**10** (Ahx-Sta)	21%	589.33	590.30	3.159	2.923	6.83 ± 0.5
**11** (Ahx-Sta-Sta)	6%	746.44	747.40	3.559	2.882	6.50 ± 0.4
**12** (Ahx-Sta-Phe)	7%	736.40	737.30	3.380	2.940	1.01 ± 0.4
**13** (Ahx-Sta-Ala)	4%	660.37	661.30	3.317	2.650	6.15 ± 0.6
**14** (Ahx-Sta-Phe-DOTA)	8%	1122.58	1123.50	3.522	3.161	1.73 ± 0.7
**15** (Ahx-Sta-Phe-αDap)	39%	822.45	823.40	3.139	2.822	2.10 ± 0.4
**16** (Ahx-Sta-Phe-Asp)	26%	851.43	852.40	3.638	3.304	1.03 ± 0.5
**17** (Ahx-Sta-Phe-Tyr)	38%	899.46	900.40	3.635	3.260	2.62 ± 0.4
**18** (Ahx-Sta-Phe-Phe)	20%	883.47	884.40	3.871	3.459	3.33 ± 0.4
**19** (Ahx-Sta-Phe-Sta)	24%	893.51	894.50	3.796	3.423	1.22 ± 0.3
**20** (Ahx-Sta-Phe-βDap)	27%	822.45	823.40	3.257	2.718	3.17 ± 0.5

Radio-HPLC chromatogram of [^{111}In]In-**22**

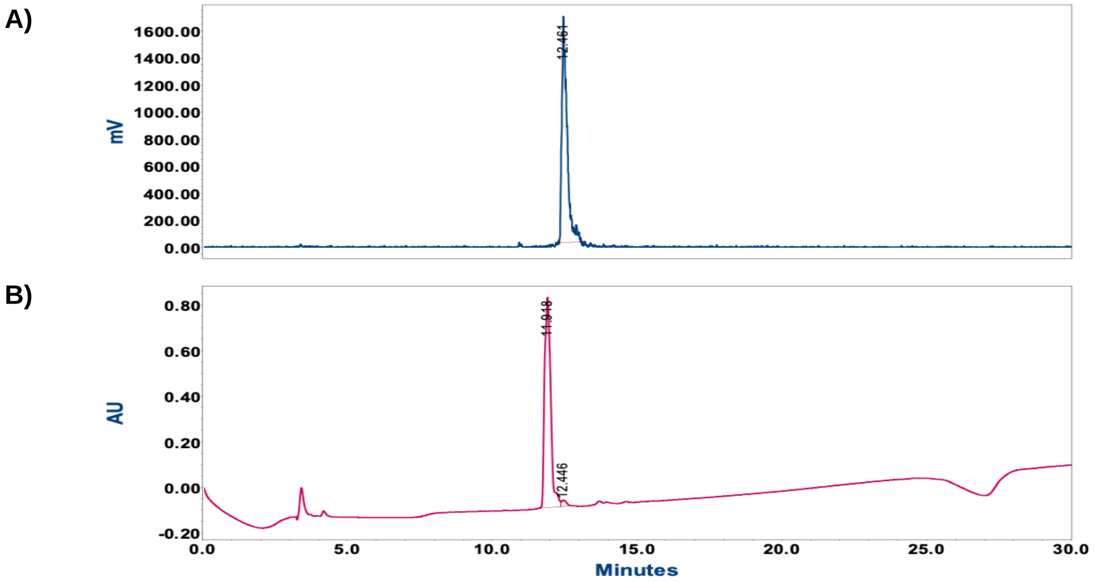

Figure S3. **A)** Radio-HPLC chromatogram of [^{111}In]In-**22**. **B)** HPLC chromatogram of **22**, visualized at 220 nM. **22** elutes at 11.91 minutes, and [^{111}In]In-**22** elutes at 12.46 minutes (RCP > 95%, chemical purity > 95%).

Radio-HPLC chromatogram of [^{111}In]In-**30**

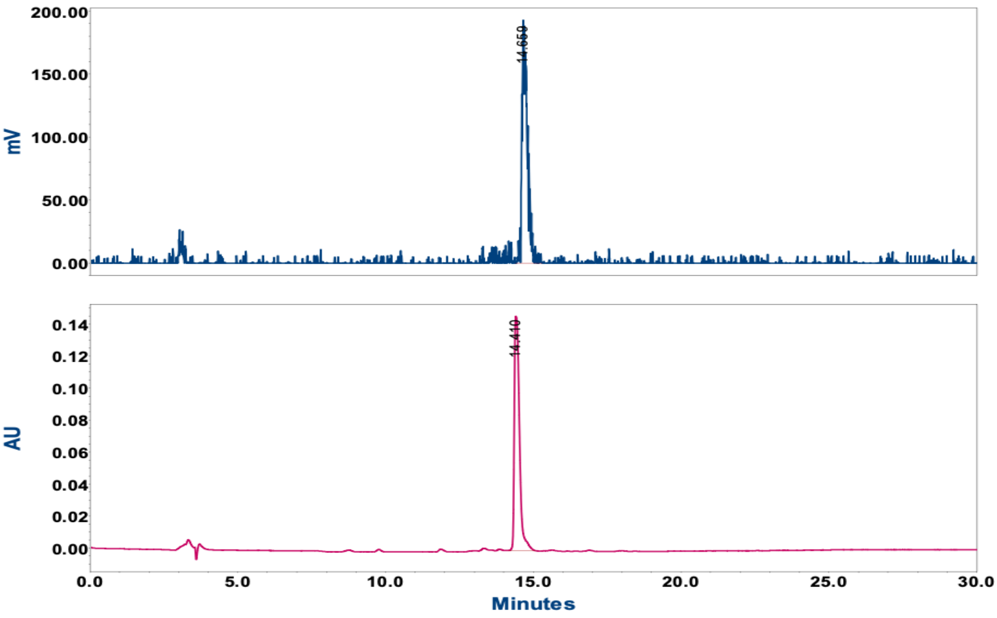

Figure S4. **A)** Radio-HPLC chromatogram of [^{111}In]In-**30**. **B)** HPLC chromatogram of **30**, visualized at 220 nM. **30** elutes at 14.41 minutes, and [^{111}In]In-**30** elutes at 14.66 minutes (RCP > 95%, chemical purity > 95%).

Radio-HPLC chromatogram of [^{111}In]In-**27**

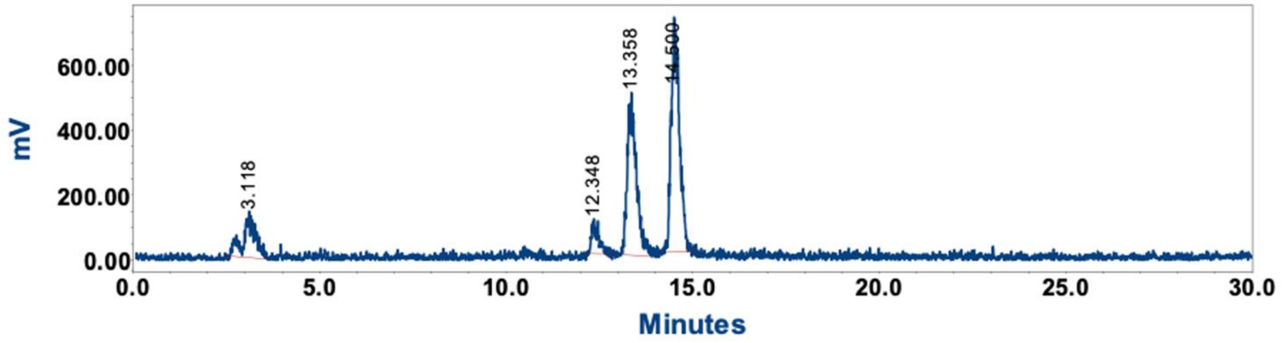

Figure S5. Radio-HPLC chromatogram of [^{111}In]In-**27** after radio-labeling. Presence of several peaks was already observed at this moment, therefore further stability studies were not performed.

Stability of [^{111}In]In-**22**

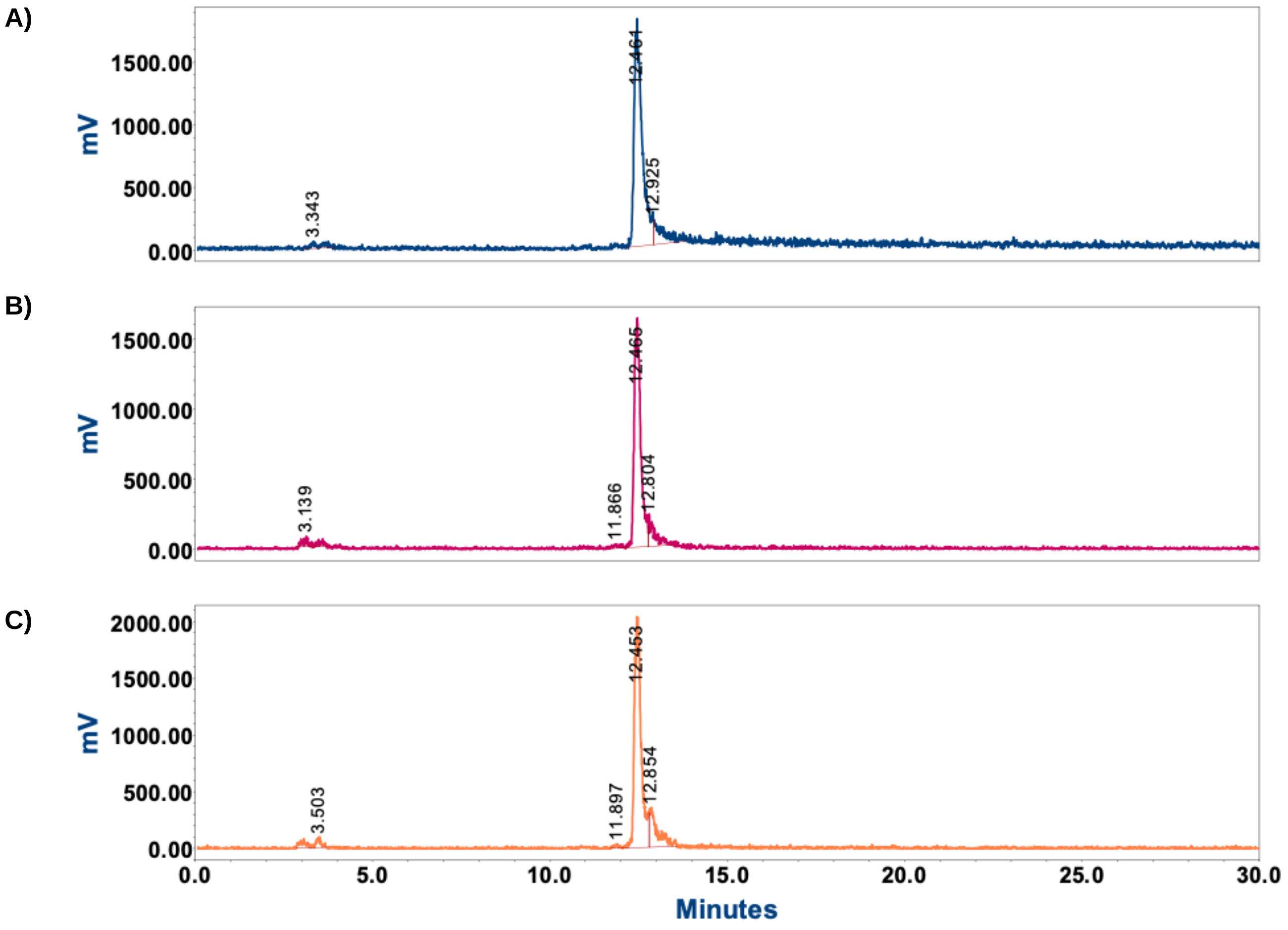

Figure S6. Radio-HPLC chromatogram of **A)** [^{111}In]In-**22**; **B)** [^{111}In]In-**22** incubated with PBS for 24 h; **C)** [^{111}In]In-**22** incubated with mouse serum for 24 h.

Stability of [^{111}In]In-**30**

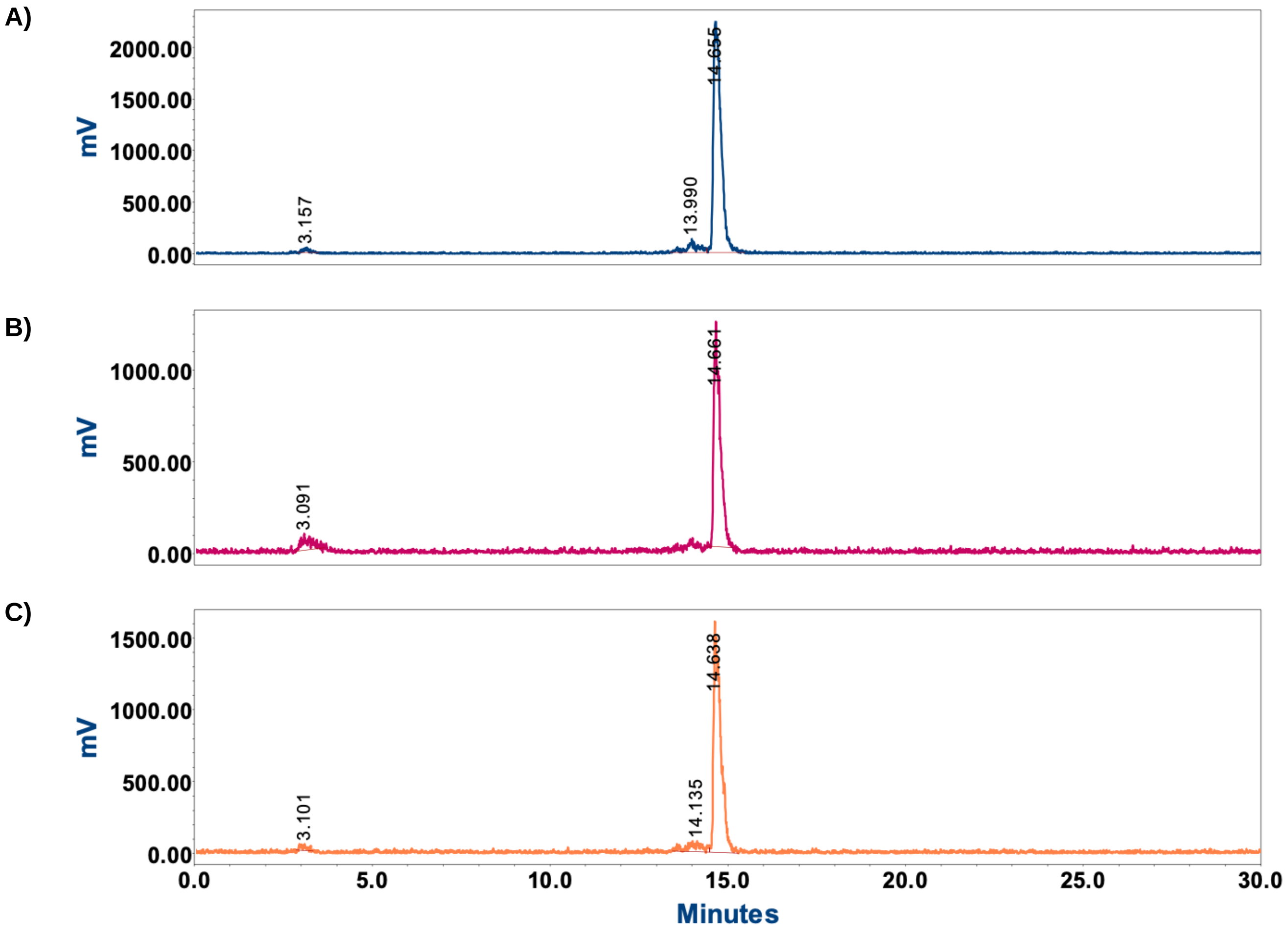

Figure S7. Radio-HPLC chromatogram of **A)** [^{111}In]In-**30**; **B)** [^{111}In]In-**30** incubated with PBS for 24 h; **C)** [^{111}In]In-**30** incubated with mouse serum for 24 h.

NAALADase Assay of PSMA-617, **22** and **30**

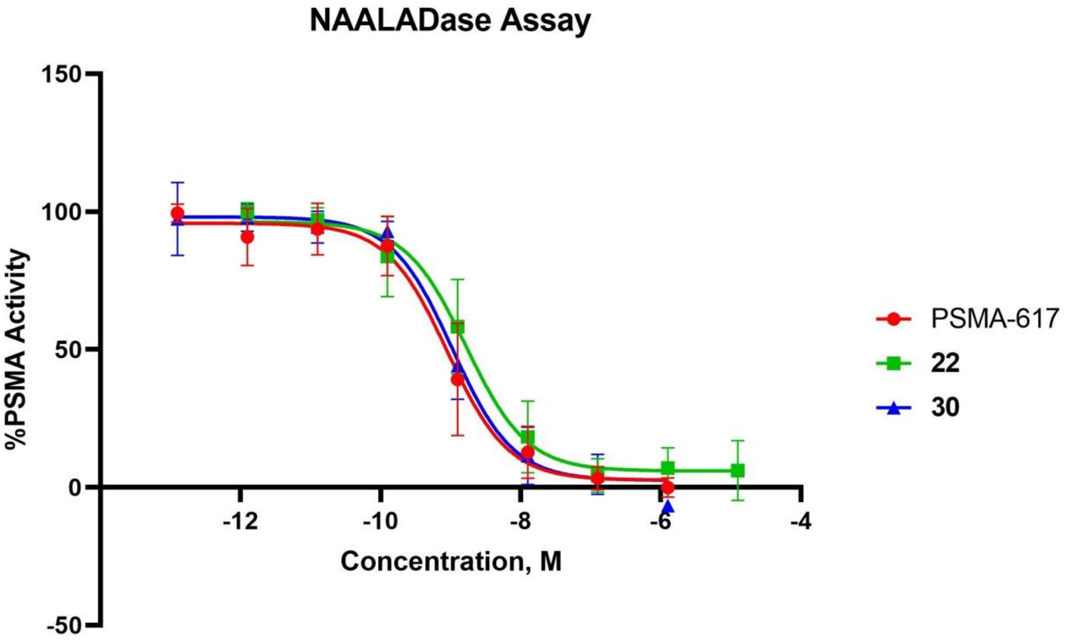

Figure S8. Competitive inhibition NAALADase assay to determine the binding affinity of compounds **22** and **30**. The compounds are incubated with the enzyme's substrate, *N*-Acetyl-Asp-Glu (NAAG), in varying concentrations.

Table S2. Numerical values of the IC_{50} obtained for PSMA-617, **22** and **30** via the NAALADase assay.

Compound	IC_{50}, nM
PSMA-617	0.90 ± 0.30
**22**	1.66 ± 0.63
**30**	1.05 ± 0.30

Uptake and internalization of [^{111}In]In-PSMA-617, [^{111}In]In-**22** and [^{111}In]In-**30** on LS174T cells

Table S3. Numerical values of the uptake and internalization of [^{111}In]In-PSMA-617, [^{111}In]In-**22** and [^{111}In]In-**30** in PSMA expressing LS174T cells.

Compound	Total uptake	Membrane-bound uptake	Internalized fraction
[^{111}In]In-PSMA-617	3.14 ± 0.47	2.07 ± 0.33 (65.9%)	1.07 ± 0.17 (34.1%)
[^{111}In]In-**22**	1.55 ± 0.21	1.02 ± 0.15 (65.8%)	0.53 ± 0.08 (34.2%)
[^{111}In]In-**30**	3.79 ± 0.74	0.26 ± 0.09 (7.4%)	3.51 ± 0.68 (92.6%)

Ex vivo biodistribution data of [^{111}In]In-PSMA-617

Table S4: *Ex vivo* biodistribution data of [^{111}In]In-PSMA-617 (20 MBq/1 nmol) at 1, 4 and 24 h post- injection (n = 4 mice/group). A block group was also performed at 4 h. Data is represented as percentage of injected dose per gram of tissue (% ID/g).

Organ	1h	4h	4h block	24h
Blood	0.96 ± 0.42	0.09 ± 0.01	0.10 ± 0.01	0.01 ± 0.00
Tumor	3.12 ± 0.30	1.59 ± 0.03	0.46 ± 0.26	0.43 ± 0.07
Heart	0.59 ± 0.23	0.07 ± 0.02	0.08 ± 0.01	0.03 ± 0.01
Lung	1.02 ± 0.35	0.14 ± 0.01	0.16 ± 0.02	0.07 ±0.01
Liver	0.74 ± 0.66	0.17 ± 0.05	0.18 ± 0.02	0.11 ± 0.03
Spleen	1.33 ± 0.40	0.23 ± 0.04	0.17 ± 0.01	0.11 ± 0.02
Stomach	0.66 ± 0.24	0.15 ± 0.05	0.19 ± 0.16	0.02 ± 0.00
Intestines	0.78 ± 0.04	0.61 ± 0.14	0.41 ± 0.13	0.06 ± 0.02
Pancreas	0.49 ± 0.29	0.08 ± 0.02	0.06 ± 0.00	0.04 ± 0.01
Kidney	14.29 ± 1.05	5.98 ± 0.95	3.90 ± 0.41	1.53 ± 0.55
Muscle	0.26 ± 0.16	0.05 ± 0.02	0.05 ± 0.00	0.02 ± 0.01
Skin	0.97 ± 0.38	0.26 ± 0.11	0.27 ± 0.06	0.12 ± 0.01
Bone	0.47 ± 0.24	0.14 ± 0.01	0.15 ± 0.02	0.11 ± 0.03
Prostate	4.49 ± 2.67	0.32 ± 0.17	0.14 ± 0.04	0.08 ± 0.06

Ex vivo biodistribution data of [^{111}In]In-**22**

Table S5: *Ex vivo* biodistribution data of [^{111}In]In-**22** (20 MBq/1 nmol) at 1, 4 and 24 h post- injection (n = 4 mice/group). A block group was also performed at 4 h. Data is represented as percentage of injected dose per gram of tissue (% ID/g).

**Organ**	**1h**	**4h**	**4h block**	**24h**
Blood	0.14 ± 0.01	0.02 ± 0.01	0.02 ± 0.00	0.00 ± 0.00
Tumor	2.05 ± 0.52	1.23 ± 0.59	0.27 ± 0.14	0.41 ± 0.08
Heart	0.13 ± 0.05	0.04 ± 0.01	0.04 ± 0.01	0.03 ± 0.01
Lung	0.45 ± 0.07	0.12 ± 0.03	0.09 ± 0.01	0.05 ± 0.01
Liver	0.17 ± 0.03	0.10 ± 0.01	0.09 ± 0.00	0.06 ± 0.01
Spleen	1.82 ± 0.37	0.30 ± 0.09	0.09 ± 0.00	0.09 ± 0.02
Stomach	0.11 ± 0.01	0.09 ± 0.06	0.17 ± 0.13	0.02 ± 0.01
Intestines	0.26 ± 0.17	0.30 ± 0.05	0.27 ± 0.03	0.04 ± 0.02
Pancreas	0.22 ± 0.03	0.06 ± 0.03	0.04 ± 0.01	0.03 ± 0.01
Kidney	22.56 ± 2.59	13.55 ± 1.04	8.20 ± 0.67	5.89 ± 1.49
Muscle	0.07 ± 0.01	0.03 ± 0.02	0.03 ± 0.01	0.03 ± 0.00
Skin	0.44 ± 0.16	0.24 ± 0.18	0.24 ± 0.07	0.15 ± 0.01
Bone	0.20 ± 0.01	0.11 ± 0.06	0.23 ± 0.13	0.13 ± 0.04
Prostate	0.51 ± 0.25	0.18 ± 0.17	0.09 ± 0.01	0.08 ± 0.03

Ex vivo biodistribution data of [^{111}In]In-**30**

Table S6: *Ex vivo* biodistribution data of [^{111}In]In-**30** (20 MBq/1 nmol) at 1, 4 and 24 h post- injection (n = 4 mice/group). A block group was also performed at 4 h. Data is represented as percentage of injected dose per gram of tissue (% ID/g).

Organ	1h	4h	4h block	24h
Blood	4.84 ± 1.44	1.02 ± 0.29	0.78 ± 0.04	0.09 ± 0.01
Tumor	4.95 ± 1.01	2.94 ± 0.36	1.42 ± 0.14	1.57 ± 0.17
Heart	2.58 ± 0.79	0.96 ± 0.22	0.82 ± 0.10	0.21 ± 0.01
Lung	2.81 ± 0.36	0.77 ± 0.52	0.84 ± 0.07	0.28 ± 0.03
Liver	3.94 ± 0.26	2.41 ± 0.35	1.84 ± 0.07	0.80 ± 0.04
Spleen	3.30 ± 0.59	1.40 ± 0.20	1.20 ± 0.14	0.58 ± 0.04
Stomach	0.47 ± 0.34	0.31 ± 0.08	0.33 ± 0.12	0.11 ± 0.07
Intestines	0.87 ± 0.08	0.92 ± 0.58	0.48 ± 0.03	0.19 ± 0.06
Pancreas	1.47 ± 0.33	0.66 ± 0.24	0.54 ± 0.07	0.17 ± 0.00
Kidney	138.54 ± 4.62	132.89 ± 11.12	120.93 ± 4.75	26.86 ± 2.37
Muscle	1.13 ± 0.22	0.64 ± 0.30	0.47 ± 0.08	0.15 ± 0.03
Skin	4.44 ± 1.06	1.92 ± 0.49	1.42 ± 0.18	0.77 ± 0.13
Bone	3.00 ± 0.53	1.44 ± 0.54	1.27 ± 0.23	0.60 ± 0.04
Prostate	3.53 ± 2.05	1.27 ± 0.69	0.94 ± 0.30	0.40 ± 0.10
